# SARS-CoV-2 infection produces an IL-33–dependent chronic eosinophilic pneumonia and muco-inflammatory airways disease in *Scnn1b-Tg* mice

**DOI:** 10.1172/jci.insight.203283

**Published:** 2026-07-07

**Authors:** Padraig E. Hawkins, Sarah R. Leist, Hong Dang, Minako Saito, Lisa C. Morton, Jesse B. Hines, Rodney C. Gilmore, Stephen A. Schworer, Ella F. Burns, Jason R. Rock, Robert S. Hagan, James J. Pestka, Alexandra Schäfer, Kenichi Okuda, Lauren K. Heine, Jack R. Harkema, Wanda K. O’Neal, Alessandra Livraghi-Butrico, Raymond J. Pickles, Ralph S. Baric, Richard C. Boucher

**Affiliations:** 1 Marsico Lung Institute/Cystic Fibrosis Research Center, School of Medicine, University of North Carolina at Chapel Hill, Chapel Hill, North Carolina, USA.; 2 Department of Epidemiology, Gillings School of Global Public Health, University of North Carolina at Chapel Hill, Chapel Hill, North Carolina, USA.; 3 Competitive Labs, Hoover, Alabama.; 4 Department of Immunology Discovery, Genentech Inc., San Francisco, California, USA.; 5 Department of Food Science and Human Nutrition and; 6 Department of Pharmacology and Toxicology, Michigan State University, East Lansing, Michigan, USA.; 7 Department of Pharmaceutical Sciences, The University of New Mexico College of Pharmacy, Albuquerque, New Mexico, USA.; 8 Department of Pathobiology and Diagnostic Investigation, Michigan State University, East Lansing, Michigan, USA.; 9 Department of Microbiology and Immunology, University of North Carolina at Chapel Hill, Chapel Hill, North Carolina, USA.

**Keywords:** Immunology, Pulmonology, Virology, COVID-19, Molecular biology, Mouse models

## Abstract

Post-acute sequelae of SARS-CoV-2 (PASC) occurs in subsets of individuals, including those with preexisting lung disease. To investigate PASC pathogenesis and therapeutics in a chronic bronchitis mouse model (*Scnn1b-*Tg), *Scnn1b*-Tg and WT mice were inoculated with a mouse-adapted SARS-CoV-2 virus (SARS-CoV-2 MA10) and followed for 60 days. Viral titer, histology, immunohistochemistry, single-cell RNA sequencing, RNA in situ hybridization, and spatial transcriptomic profiling characterized disease pathologies. *Scnn1b*-Tg mice inoculated with SARS-CoV-2 MA10 exhibited lower viral titers and less weight loss than WT mice. Airway epithelia of *Scnn1b*-Tg mice were less infected than epithelia of WT mice, reflecting increased airway mucus and enhanced epithelial antiviral activities in *Scnn1b-*Tg mice. However, *Scnn1b*-Tg mice subsequently exhibited heterogeneous airway and parenchymal disease with elevated *Il33* expression characteristic of human eosinophilic pneumonia. Cohorts of infected mice were given a monoclonal antibody targeting the IL-33 receptor (ST2) or enteral prednisone. Administration of an anti-ST2 monoclonal antibody mitigated development of eosinophilic pneumonia, while enteral prednisone suppressed IL-33 expression and disease. The eosinophilic pneumonia in *Scnn1b*-Tg mice after SARS-CoV-2 MA10 infection mimics reports of eosinophilic pneumonia in humans after SARS-CoV-2, suggesting that targeting of IL-33 may be beneficial in treating post-viral eosinophilic pneumonia in humans.

## Abstract 2

Post-acute sequalae of COVID-19 causes significant morbidity. Our study in mice provides insights into both mechanisms and potential therapeutic strategies of post-acute sequalae of COVID-19.

## Introduction

Severe acute respiratory syndrome coronavirus 2 (SARS-CoV-2) emerged in late 2019 and caused the coronavirus disease 2019 (COVID-19) pandemic. Management of acute COVID-19 has been greatly improved by the development of vaccines and novel therapeutics (1, 2). These advances, coupled with the emergence of new SARS-CoV-2 variants with reduced distal lung growth kinetics (3) and community immunity, have resulted in lower acute morbidity and mortality associated with COVID-19 than initially reported (4). However, the estimated global, cumulative incidence of post-acute sequelae of SARS-CoV-2 infection (PASC), as defined by multisystemic (neurologic, cardiovascular, pulmonary, metabolic, and immunologic) complications in infected populations, is approximately 400 million (5), suggesting that PASC remains a serious problem worldwide (6). Pulmonary PASC is heterogeneous, spanning organizing pneumonia-like remodeling, small-airways disease with mucus plugging, fibrotic scarring, pulmonary vascular complications, and nonstructural exercise-limitation phenotypes (7–12).

Among all patients hospitalized with COVID-19, about 11% of those discharged exhibited residual lung abnormalities by computed tomography (CT) within 8 months of discharge (13). Ground-glass opacities or reticulation findings consistent with chronic organizing pneumonia were more prevalent than usual interstitial pneumonia (UIP) patterns. More rarely and perhaps reflecting the requirement of bronchoalveolar lavage for diagnosis, eosinophilic pneumonia was also reported as part of the PASC syndrome (14–16). In addition to the various pulmonary PASC endotypes, it has been reported that individuals with preexisting respiratory diseases, including chronic obstructive pulmonary disease (COPD), asthma, bronchiectasis, and interstitial lung disease (ILD), are at higher risk for PASC (17). However, identification of lung disease–specific PASC endotypes and therapies remains elusive.

Murine models based on common mouse strains and mouse-adapted SARS-CoV-2 have been developed that mimic the post-infectious, fibrotic ILD observed in a subset of PASC (18). Given the heterogeneity of pulmonary PASC, and its likely dependence on preexisting lung disease, we hypothesized that murine models with underlying lung disease could yield novel phenotypes relevant to the spectrum of human PASC manifestations and provide mechanisms to inform tailored countermeasures. To this end, we characterized the short- and long-term pulmonary phenotypes in a mouse model of chronic bronchitis (*Scnn1b*-Tg) infected with a mouse-adapted SARS-CoV-2 strain (MA10) (19, 20). Acute and chronic lung disease in *Scnn1b*-Tg mice and matched wild-type (WT) controls was investigated using histology, immunohistochemistry (IHC), viral and immunologic assays, spatial profiling of the whole transcriptome, and RNA in situ hybridization (RNA-ISH). Cumulatively, our data indicate that *Scnn1b*-Tg mice developed post–COVID-19 eosinophilic pneumonia and identify the IL-33 signaling pathway as a therapeutic target for mitigating this PASC endotype.

## Results

### Scnn1b-Tg mice exhibit reduced mortality, weight loss, and titers after SARS-CoV-2 MA10 infection compared with WT mice, but Scnn1b-Tg mice develop chronic pulmonary lesions.

**Figure 1:**
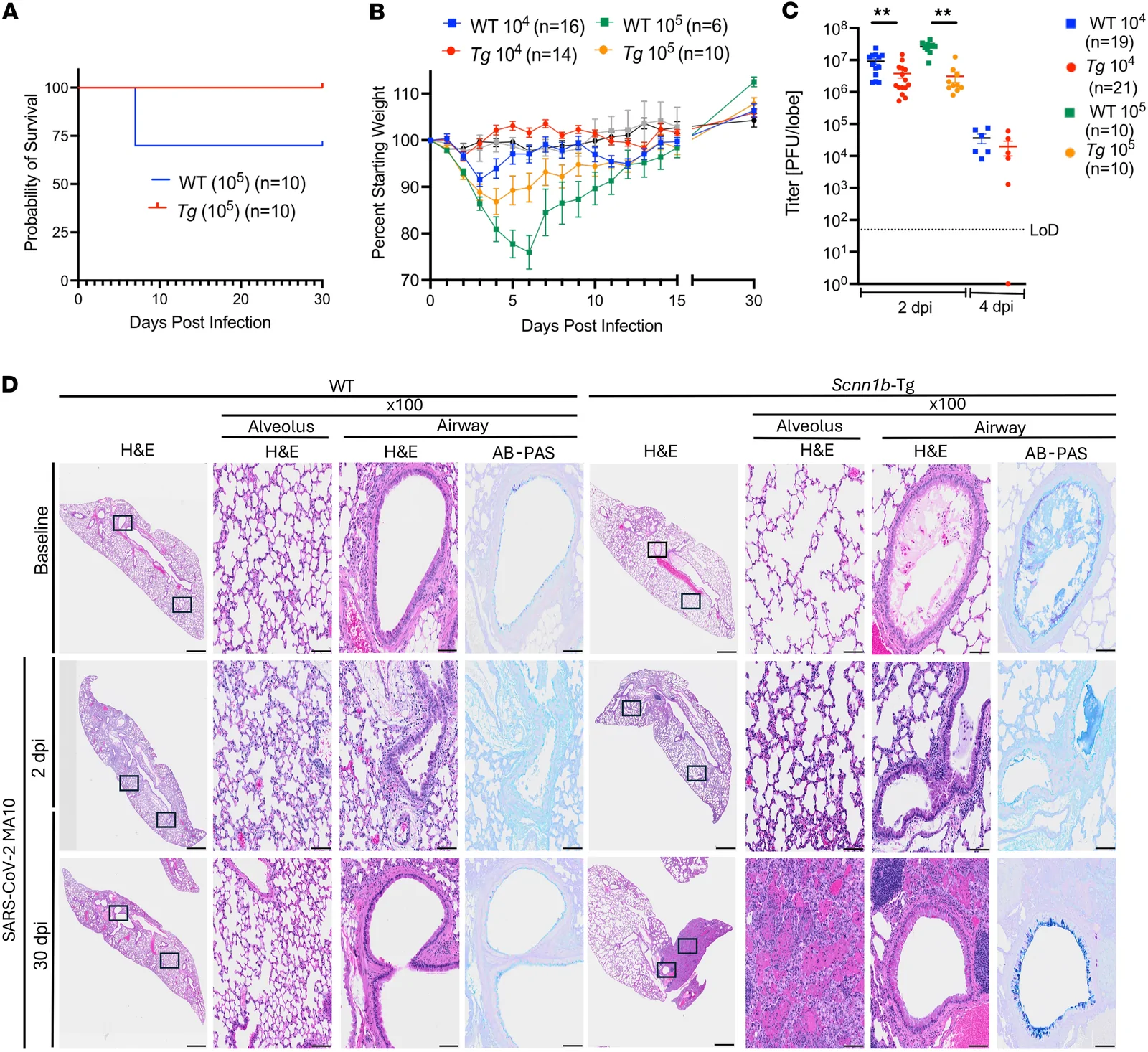
*Scnn1b*-Tg mice are protected from acute illness after SARS-CoV-2 MA10 but develop chronic disease. (**A**) Survival score with 10^5^ PFU. (**B**) Percentage starting weight across time (dpi) for indicated experimental groups. Data were analyzed by mixed-effects analysis followed by Šidák’s multiple comparisons. At 10^4^ PFU there was a statistically significant difference at 3 and 4 dpi; at 10^5^ PFU there was a significant difference at 6 dpi. (**C**) Infectious virus lung titer as determined by plaque assay at either 2 dpi (2 viral inocula, 10^4^ or 10^5^ PFU) or 4 dpi (10^4^ PFU dose only) in wild-type (WT) or *Scnn1b*-Tg mice. Dotted line represents the limit of virus detection (LoD). Error bars represent SEM. PFU, plaque-forming units. (**D**) Histopathologic analysis of lungs at baseline and at indicated times dpi. H&E, hematoxylin and eosin. “AB-PAS” indicates Alcian blue/periodic acid–Schiff staining with mucins appearing dark blue. Scale bars: 1 mm (low magnification) and 50 μm. Mixed-effects analysis followed by Šidák’s multiple comparisons. ANOVA followed by Kruskal-Wallis test. **P < 0.01.

The 10^4^ plaque-forming unit (PFU) inoculum produced no mortality in *Scnn1b*-Tg or WT mice, while 10^5^ PFU produced 33% mortality selectively in the WT mice (Figure 1A). *Scnn1b*-Tg mice experienced less body weight loss and returned to starting body weight more quickly than WT, with both WT and *Scnn1b*-Tg mice returning to baseline weights at 30 days post-inoculation (dpi) (Figure 1B). From previous studies, WT (C57BL/6J) mice inoculated with SARS-CoV-2 MA10 typically reach peak viral lung titers 2 days after inoculation (21). Notably, viral titers were lower in *Scnn1b*-Tg mouse lungs at 2 dpi than in WT mice inoculated with both 10^4^ and 10^5^ PFU (Figure 1C). By 4 dpi, viral titers dropped in both *Scnn1b*-Tg and WT infected with 10^4^ PFU and became similar (Figure 1C). Gross lung congestion scores for the 10^4^ and 10^5^ PFU inoculum cohorts were not different in WT versus *Scnn1b*-Tg mice at 2 and 4 dpi (Supplemental Figure 1A; supplemental material available online with this article; https://doi.org/10.1172/jci.insight.203283DS1).

Histologic features characteristic of the *Scnn1b*-Tg model, e.g., alveolar enlargement, increased airway Alcian blue/periodic acid–Schiff–positive (AB-PAS^+^) staining, and airway mucus plugging (20), were evident at baseline in *Scnn1b-*Tg mice (Figure 1D). Both WT and *Scnn1b-*Tg mice shared evidence of immune cell infiltration in the airway and parenchyma, alveolar congestion, and epithelial damage at 2 dpi (Figure 1D and Supplemental Figure 1, B and C). By 30 dpi, WT mice inoculated with 10^4^ PFU MA10 showed no gross lung abnormalities (Figure 1D). However, 40% of *Scnn1b*-Tg mice exhibited histopathologic abnormalities in both airway and parenchymal regions (Figure 1D). The parenchymal lesions were characterized by an accumulation of hyper-eosinophilic intra-alveolar macrophages, hyperplastic alveolar epithelial cells, eosinophils, chitinase crystals, and Masson bodies (Supplemental Figure 2, A–E, and Supplemental Figure 3). The conducting airways proximal to parenchymal lesions exhibited evidence of goblet cell metaplasia, with increased intracellular mucins, as detected by AB-PAS staining (Figure 1D).

### Scnn1b-Tg airway epithelium exhibits reduced SARS-CoV-2 infection.

**Figure 2:**
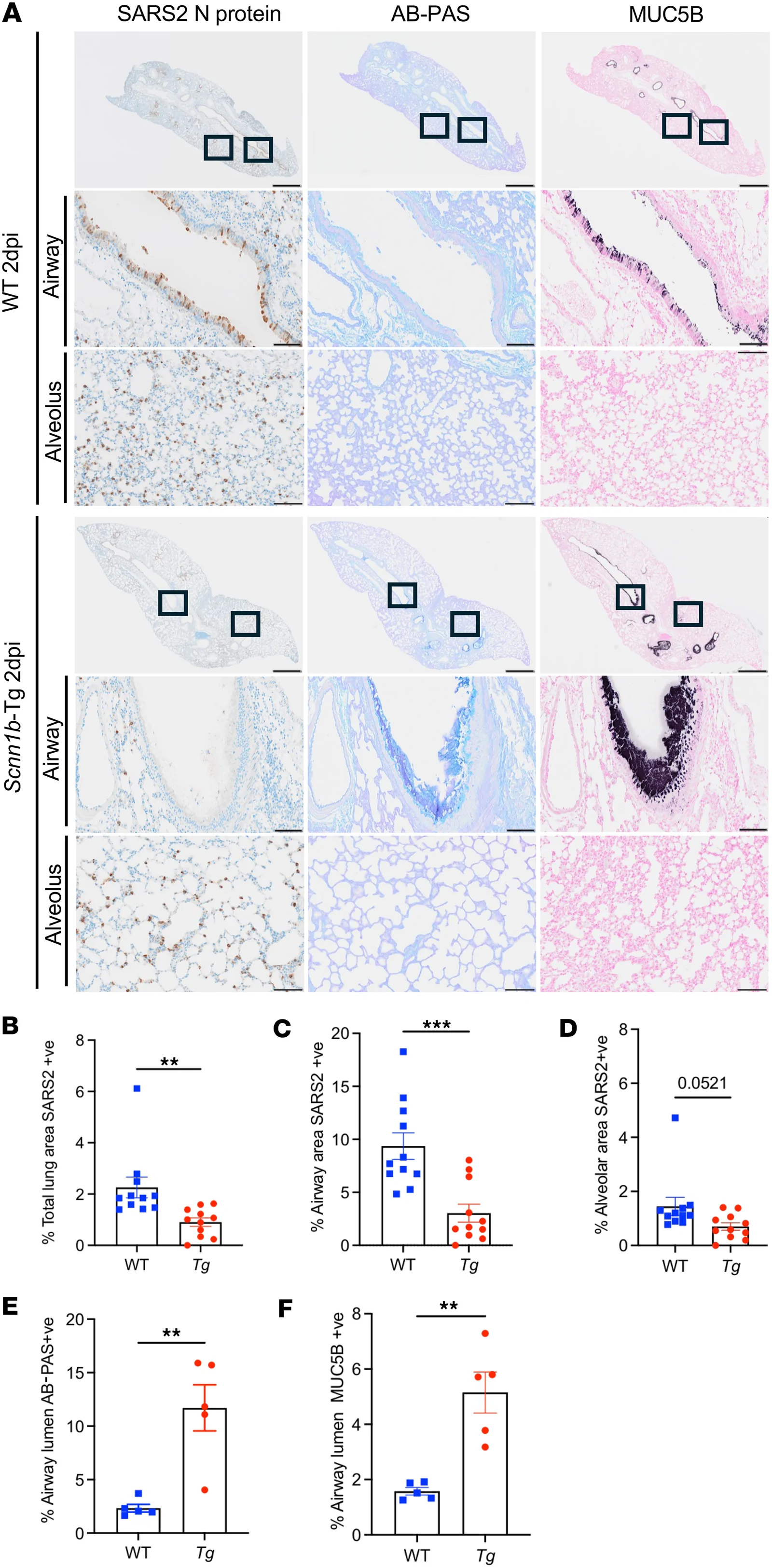
Airway epithelium of *Scnn1b*-Tg mice is resistant to infection with SARS-CoV-2 MA10. (**A**) Representative images of lungs from WT and *Scnn1b*-Tg mice at 2 dpi with 10^4^ PFU of SARS-CoV-2 MA10. Shown are areas of alveolus and airway (labeled). Shown are IHC against SARS-CoV-2 nucleocapsid (N) protein and AB-PAS staining of mucins and IHC against MUC5B. Scale bars: 1 mm (low magnification) and 50 μm. (**B**–**D**) IHC of SARS-CoV-2 nucleocapsid protein was quantitated and normalized to lung tissue area for whole lung (**B**), airway (**C**), and alveolar tissue (**D**). (**E** and **F**) Airway lumen mucus was quantified using AB-PAS (**E**) and IHC of MUC5B (**F**) expressed as a percentage of the entire lumen area. Each dot represents results for one individual animal. **P < 0.01; ***P < 0.001. Student’s *t* test.

The tropism of MA10 for respiratory epithelia in mice has been previously described, with airway secretory cells and alveolar type 2 (AT2) cells exhibiting the greatest susceptibility to infection (22). In our studies, virus was detected in the airway and alveolar compartments of both WT and *Scnn1b*-Tg mice at 2 dpi after inoculation with 10^4^ PFU (Figure 2A and Supplemental Figure 1, B and C). However, consistent with lower viral titers in *Scnn1b*-Tg mice (Figure 1C), morphometric quantification of virus infection by IHC for SARS-CoV-2 nucleocapsid (N) protein and RNA-ISH for spike mRNA revealed fewer viral particles in the lungs of *Scnn1b*-Tg versus WT (Figure 2, A–D, and Supplemental Figure 1, B and C). A 3-fold lower value of SARS-CoV-2 N protein was measured in the airways of *Scnn1b*-Tg mice at 2 dpi, with alveolar infection trending lower in the *Scnn1b*-Tg versus WT (Figure 2, B–D). The lower levels of SARS-CoV-2 staining in the *Scnn1b*-Tg mice were accompanied by preservation of secretory and AT2 cell markers (Scgb1a1 and Sftpc, respectively) in comparison with WT littermates, which exhibited a significantly reduced number of *Sftpc^+^* AT2 cells upon MA10 infection (Supplemental Figure 4, A–C) (21).

### Accumulated proximal airway mucus in Scnn1b-Tg mice is associated with reduced airway epithelial SARS-CoV-2 infection.

Because *Scnn1b*-Tg mice accumulate Muc5b-predominant mucus on proximal airway surfaces (23), a simple hypothesis to explain lower viral titers in *Scnn1b*-Tg mouse airways is the increased barrier protection provided by hyperconcentrated/adherent mucus (Figure 2A). Quantitation of mucus accumulation in *Scnn1b*-Tg versus WT mice revealed an increase in airway AB-PAS and Muc5b staining in *Scnn1b*-Tg mice (Figure 2, E and F). Notably, areas of airway epithelium underlying mucus plugs/plaques were predominantly free of viral antigen staining (Figure 2A). In contrast, bronchoalveolar duct junctions (BADJs) did not exhibit accumulation of mucus, and in this region there were no genotype-dependent differences in SARS-CoV-2 infection, and evidence of infection was obvious with a marked accumulation of inflammatory cells and epithelial cell debris in the airways of both WT and *Scnn1b*-Tg mice at 2 dpi (Supplemental Figure 1).

### Baseline activation of proximal airway epithelial antiviral pathways in Scnn1b-Tg mice also associates with reduced SARS-CoV-2 infection.

To further elucidate how the muco-inflamed airways of *Scnn1b-*Tg mice may limit MA10 infection, we investigated whether the airway epithelium of *Scnn1b*-Tg mice may be primed to inhibit MA10 infection. The presence of adherent airway mucus has been previously shown to trigger epithelial responses, e.g., activation of epithelial inflammatory pathways and immune cell chemotaxis, likely via retention of microbes, toxins, allergens (24), and/or epithelial hypoxia (25). Activation of these pathways could produce innate antiviral activities that contribute to decreased MA10 infectivity in the airways of *Scnn1b*-Tg mice versus WT mice.

To capture gene expression changes due to baseline differences and/or viral challenge, digital spatial profiling (DSP) was performed in selected cohorts of naive (baseline) and infected (2 dpi and 30 dpi) WT and *Scnn1b*-Tg mice (Supplemental Figure 5A). Regions of interest (ROIs) included proximal airway epithelia (defined as the mainstem bronchus to the first divisions of the intrapulmonary bronchi), distal BADJ regions, and alveolar parenchyma (diseased [chronic lesions] and non-diseased [normal-appearing histology]) (Supplemental Figure 5B). Note that all ROIs were collected in a single batch, but presentation of the data is considered by airway region, infection status, and time after infection. All DSP data used for each analysis are available in Supplemental Data File 1.

**Figure 3:**
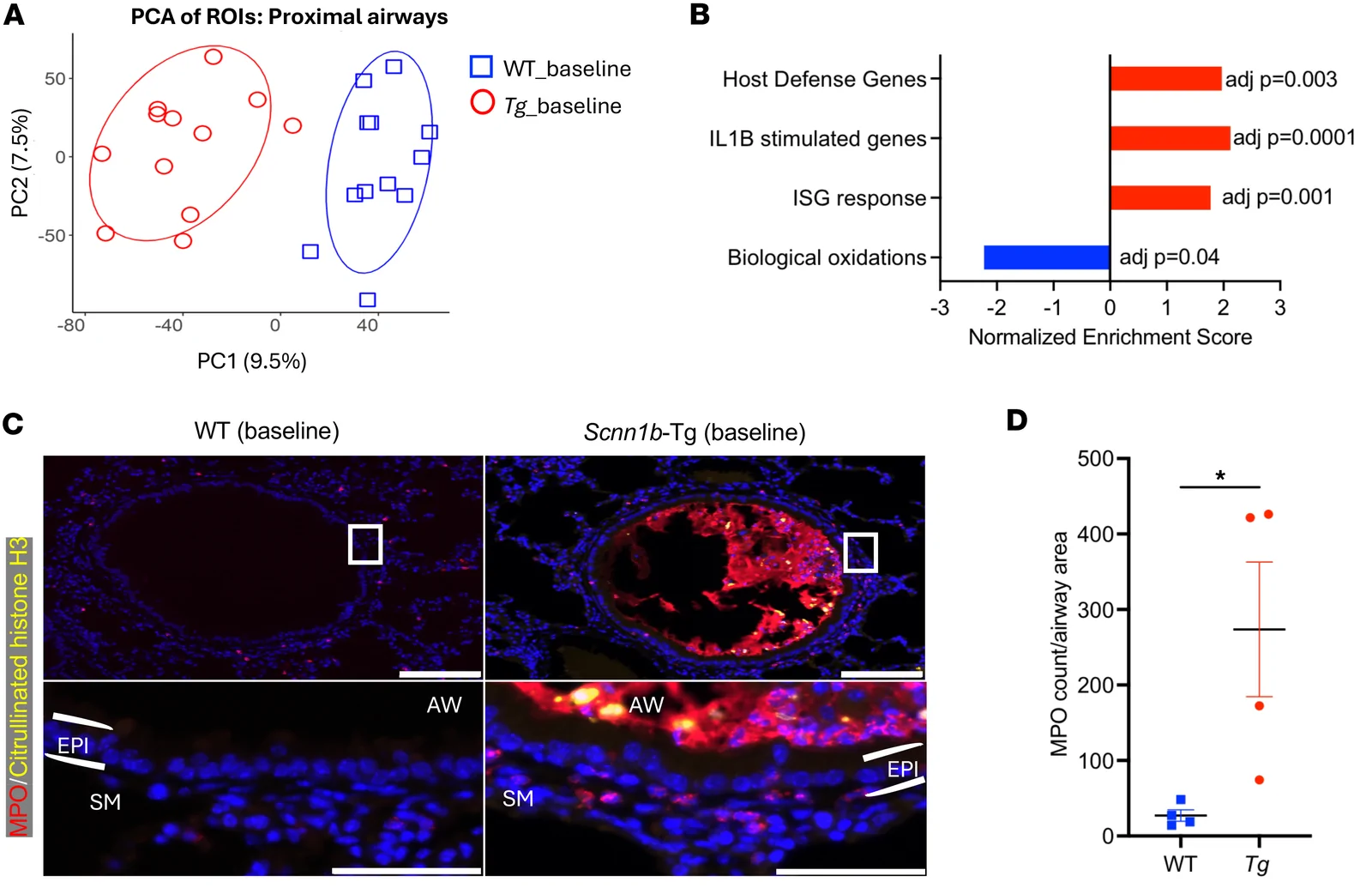
Airway epithelium of *Scnn1b*-Tg mice shows epithelial and immune priming that is protective against SARS-CoV-2 MA10. (**A**) PCA plot of gene expression values from spatial transcriptomics of proximal airway regions of interest (ROIs). All ROIs selected are displayed; red circles represent baseline plugged proximal airways of *Scnn1b*-Tg mice, and blue squares represent baseline proximal airways of WT mice. (**B**) Reactome and custom gene set enrichment analysis shown as normalized enrichment score of baseline *Scnn1b*-Tg-plugged versus baseline WT proximal airways. (**C**) Immunofluorescence for myeloperoxidase (MPO) and citrullinated histone H3 is shown in baseline, non-infected WT and *Scnn1b*-Tg-plugged mouse airways. Bottom panels represent higher magnification of inset. Scale bars: 100 μm and 50 μm (higher magnification). (**D**) Plot shows quantitation of MPO^+^ cells using immunofluorescence, displayed as numbers of cells per mm^2^ of airway. Each dot shows results for one individual animal. EPI, epithelium; AW, airway lumen; SM, submucosa. *P < 0.05. Student’s *t* test.

We first evaluated the baseline (pre-virus/naive) proximal airways where mucus plugs are found in the *Scnn1b*-Tg mice versus WT mice. Principal component analysis (PCA) of proximal airway ROIs at baseline (WT = not plugged vs. *Scnn1b*-Tg = plugged) showed distinct separation between genotypes (Figure 3A), with many differentially expressed genes (DEGs) (Supplemental Data File 2). Pathway analyses of DEGs detected upregulation of IFN-stimulated genes (ISGs), *Il1b*-stimulated genes, and host defense, concurrent with downregulated biological oxidation pathways, in *Scnn1b-*Tg mice as compared with WT mice (Figure 3B and Supplemental Data File 3). Upregulated ISG epithelial genes reported to be protective against SARS-CoV-2 infection included *Serping1* and *Ifitm1* (Supplemental Figure 6C) (26). Similarly, several epithelial host defense genes were upregulated in the proximal airways of *Scnn1b*-Tg mice that may also confer antiviral activities either directly, e.g., *Plscr1* and *Lcn2*, or indirectly, e.g., *Cd177* (activated neutrophil marker) (Supplemental Figure 6C) (27).

### Baseline proximal airway luminal neutrophil accumulation is also associated with reduced SARS-CoV-2 infection in Scnn1b-Tg mice.

We next considered whether the antiviral activities present in the proximal airways of the *Scnn1b*-Tg mice might also reflect the presence of inflammatory cells within the epithelium captured by the ROIs. Using immunofluorescence for neutrophil myeloperoxidase (MPO) and citrullinated histone H3, an increased number of neutrophils and neutrophil extracellular trap formations was observed within the airway epithelium of *Scnn1b*-Tg mice at baseline (Figure 3, C and D), consistent with previous reports (28, 29). Therefore, the increased abundance of neutrophils, which also express *Ifitm1* (Human Protein Atlas; www.proteinatlas.org) (30), may also contribute to the reduced infection of the airway epithelium of *Scnn1b*-Tg mice with MA10 (31).

### Early (2 dpi) transcriptional responses of proximal airways to MA10 infection are qualitatively similar and virus infection–proportionate in Scnn1b-Tg mice and WT mice.

While baseline differences in the proximal airways of *Scnn1b-*Tg and WT mice were significant as described above (Figure 2D), PCA analysis indicated that the proximal airways of WT and *Scnn1b*-Tg mice exhibited similar transcriptional responses to MA10 infection at 2 dpi (Supplemental Figure 6A), largely driven by strong upregulation of ISGs, cytokine signaling, cell senescence, and complement cascade genes and decreases in biological oxidations (Supplemental Figure 6B). Notably, while still robust, the ISG response in the proximal airways of *Scnn1b*-Tg mice was overall reduced in comparison with WT mice (Supplemental Figure 6D), likely reflecting the lower viral burden as measured by titers (Figure 1C).

### BADJ regions in Scnn1b-Tg and WT mice at baseline and 2 dpi.

In contrast to the proximal airways, ROIs selected from BADJs, which were not mucus-plugged in the *Scnn1b*-Tg mice, showed minimal transcriptional differences between genotypes both at baseline and at 2 dpi (Supplemental Figure 7).

### Airway mucous cell metaplasia with Th2 skewing develops in MA10-infected Scnn1b-Tg mice in regions with evidence of chronic disease at 30 dpi.

**Figure 4:**
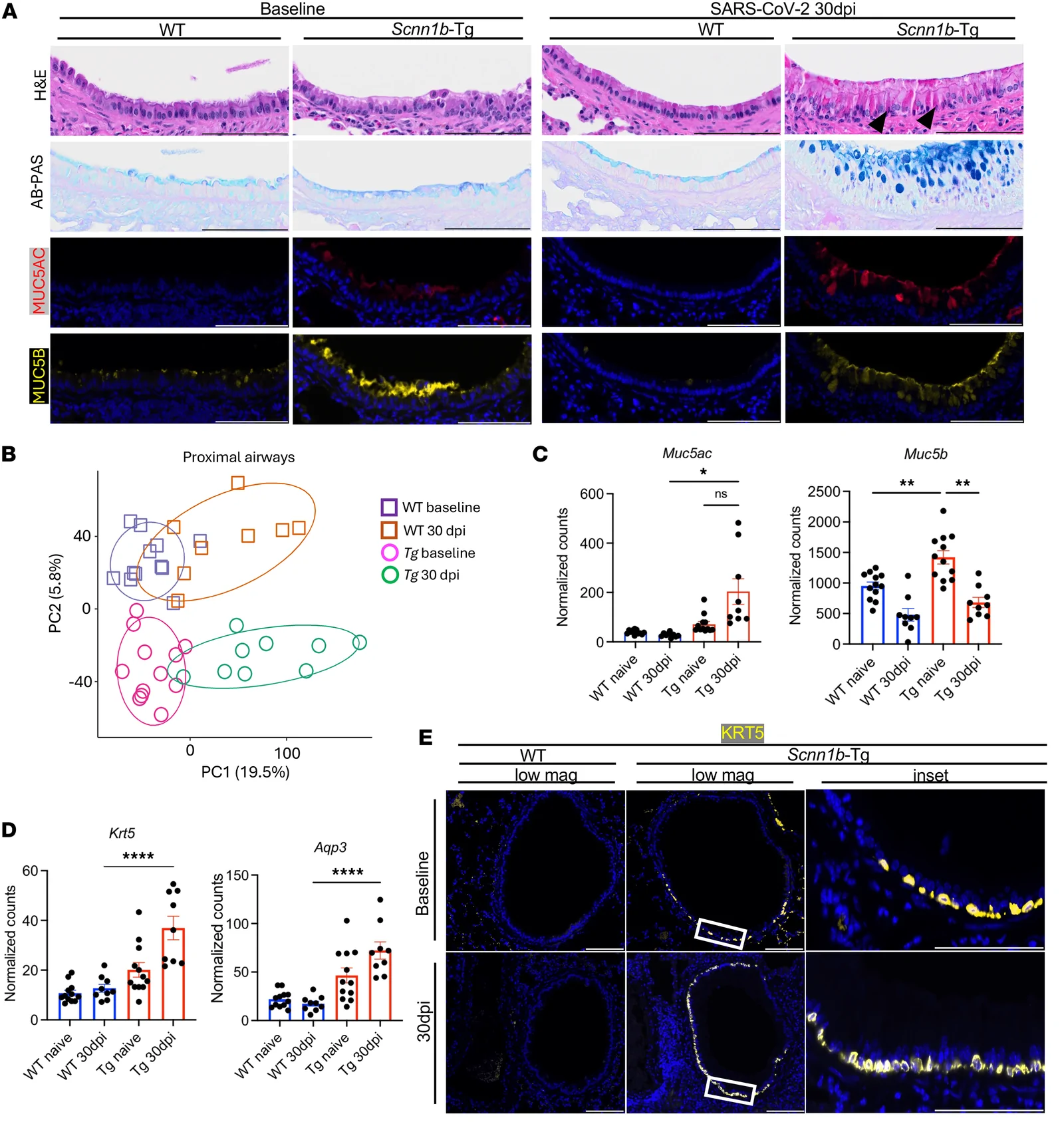
SARS-CoV-2 MA10 infection induces MUC5AC-rich goblet cell metaplasia in the airways of *Scnn1b*-Tg mice at 30 dpi. (**A**) Histopathologic analysis of WT and *Scnn1b*-Tg mouse airways in non-infected mice (baseline) and at 30 dpi. MUC5AC and MUC5B are visualized after IHC. H&E, hematoxylin and eosin; AB-PAS, Alcian blue/periodic acid–Schiff with mucins appearing dark blue. Black arrowheads show intracellular YM1/YM2. Scale bars: 100 μm. (**B**) PCA plot of gene expression values from GeoMx spatial transcriptomics of proximal airways is shown for the indicated experimental groups. All ROIs selected are displayed, with each color representing an experimental group and symbols representing genotype. (**C**) Q3-normalized counts representing RNA expression of *Muc5ac* and *Muc5b* across proximal airway ROIs in WT and *Scnn1b*-Tg mice for naive and 30 dpi. (**D**) Q3-normalized counts representing gene expression of *Aqp3* and *Krt5* across proximal airway ROIs in WT and *Scnn1b*-Tg mice for naive (baseline) and 30 dpi. (**E**) IHC for KRT5 in representative airways of WT and *Scnn1b*-Tg mice, naive and at 30 dpi. Scale bars: 100 μm for all. Each dot in bar plots of **C** and **D** shows results for one individual animal. *P < 0.05; **P < 0.01; ****P < 0.0001. ANOVA with Tukey’s multiple-comparison test.

Histologically, airways of WT mice recovered from the acute lung injury by 30 dpi (Figure 1D and Figure 4A). In contrast, a proportion (40%) of *Scnn1b*-Tg mice exhibited regions of goblet cell metaplasia, which were typically within, or proximal to, the parenchymal lesions identified in *Scnn1b*-Tg mice at 30 dpi (Figure 1D and Figure 4A). In these regions, airway epithelia were hyperplastic with characteristic AB-PAS^+^ goblet cells with increased MUC5AC and decreased MUC5B expression as assessed by immunofluorescence (Figure 4A).

PCA analysis comparing WT and *Scnn1b*-Tg proximal airways at baseline or at 30 dpi identified separation of 30 dpi from baseline for both WT and *Scnn1b*-Tg, suggesting incomplete recovery from infection for both cohorts (Figure 4B). We specifically focused on the characteristics of the mucus metaplastic lesions in the *Scnn1b*-Tg mice. As visualized in a heatmap of DEGs, genes belonging to cluster 2 were uniquely upregulated in diseased *Scnn1b*-Tg airways at 30 dpi (Supplemental Figure 6B and Supplemental Data File 2). This cluster included *Muc5ac* (Figure 4C), genes related to increased mucin secretion (e.g., *Ern2*), and genes associated with chitinase production (*Chil3*/*Chil4*) (Supplemental Figure 8A), supporting the hypothesis that a type 2 inflammatory stimulus triggered mucous secretory cell metaplasia. As predicted from the IHC, MUC5B was upregulated in these diseased airways (Figure 4C). RNA-ISH confirmed upregulation of the chitinases *Chil3* and *Chil4* and colocalized their expression with the airway secretory cell marker *Scgb1a1* (Supplemental Figure 8B).

*Krt5* and *Aqp3*, markers of airway basal cells, were also upregulated in *Scnn1b*-Tg airways compared with WT at 30 dpi (Figure 4D and Supplemental Data File 1), indicating an epithelial proliferative response. Validating this finding, IHC identified more KRT5^+^ basal cells in *Scnn1b*-Tg compared with WT proximal airways at baseline, and the KRT5^+^ population was even more abundant in the diseased airways of *Scnn1b*-Tg mice at 30 dpi (Figure 4E).

### MA10 infection results in development of morphologic features of chronic (30 dpi) organizing eosinophilic pneumonia in Scnn1b-Tg mice.

**Figure 5:**
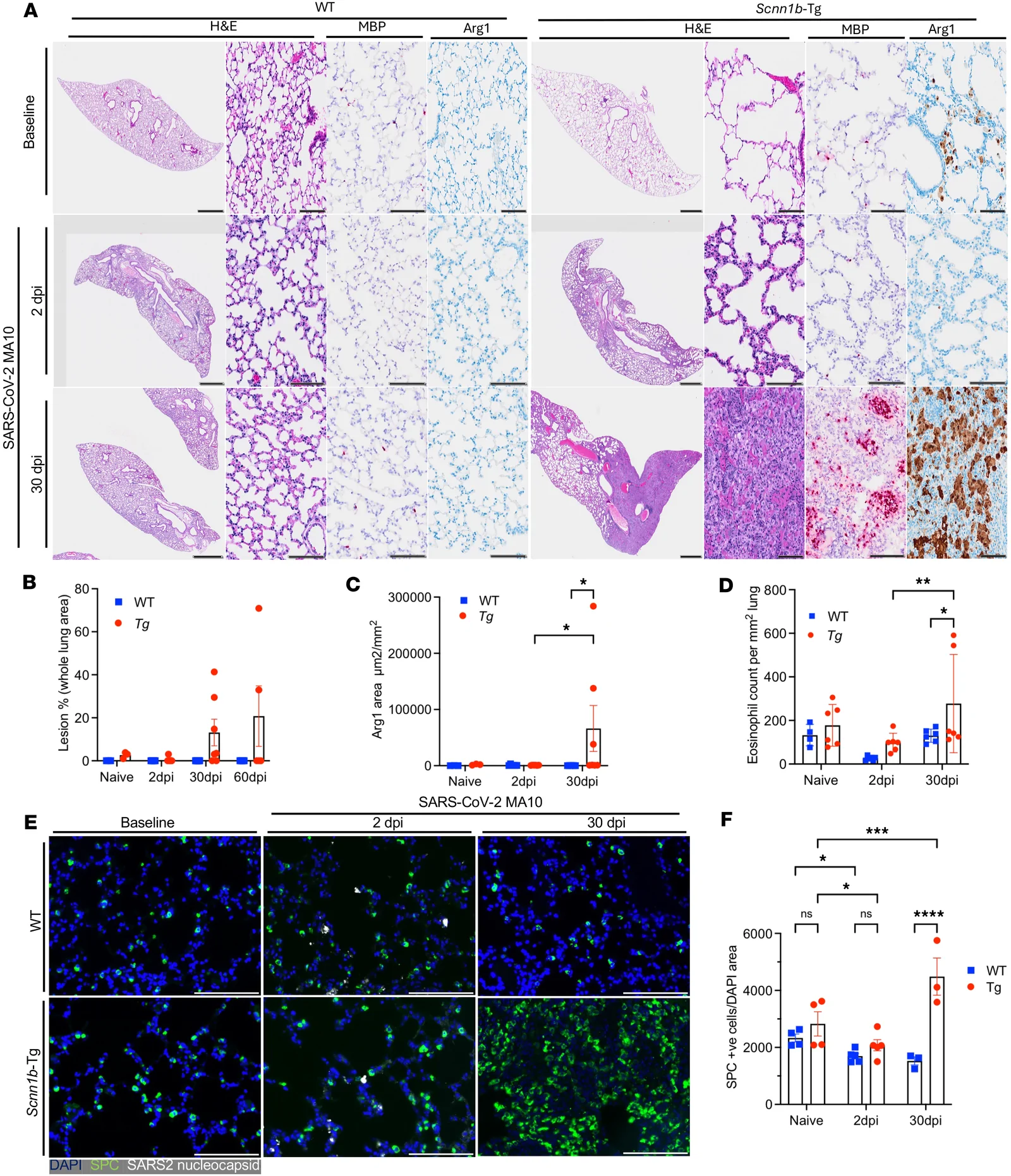
SARS-CoV-2 MA10 infection causes variable levels of chronic eosinophilic pneumonia in *Scnn1b*-Tg mice. (**A**) Histopathologic analysis of lungs in the experimental groups (non-infected = baseline, 2 and 30 dpi for WT and *Scnn1b*-Tg mice). H&E, hematoxylin and eosin. MBP indicates IHC against major basic protein, a marker of eosinophils. Arg1 indicates IHC against arginase-1, a marker of M2 macrophage activation. Scale bars: 1 mm (low magnification) and 50 μm. (**B**) Quantitation of the percentage of lung tissue affected by chronic eosinophilic pneumonia. (**C**) Arg1 area quantified and normalized to lung area. (**D**) Eosinophil count based on IHC against MBP and normalized to whole lung area. (**E**) Immunofluorescence of pro–surfactant protein C and SARS-CoV-2 nucleocapsid protein shown over a time course of infection in WT and *Scnn1b*-Tg mice. DAPI staining nuclei in dark blue. Scale bars: 100 μm. (**F**) Quantitation of pro–surfactant protein C–positive cells per total DAPI area. Dots in bar graphs represent individual animals (**B**–**D** and **F**). *P < 0.05; **P < 0.01; ***P < 0.001; ****P < 0.0001. Mixed-effects analysis, uncorrected Fisher’s least significant difference.

The 30 dpi airway data identified a Th2-skewed response associated with the chronic lesions. A similar investigation of the parenchymal lesions was conducted to explore the mechanisms mediating lesion development (Figure 5A). The lesions, which varied in size (Figure 5B), were shown to contain major basic protein (Figure 5, A and D), which marked eosinophils, and the large hyper-eosinophilic macrophages (Supplemental Figures 2 and 3) were shown to contain ARG1, a marker of alternative macrophage activation (Figure 5, A and C). Both eosinophils and alternatively activated macrophages persisted in these lesions at 60 dpi (Supplemental Figure 9A). The lesion also demonstrated fibroblast proliferation, as measured by IHC for α-smooth muscle actin (α-SMA), and focal collagen deposition, as indexed by increased Picrosirius red histochemical stain and COL1A1 IHC, consistent with remodeling of alveolar structures in these lesions (Supplemental Figure 9B). α-SMA–stained myofibroblasts occasionally accumulated within the alveolar spaces or terminal bronchioles reminiscent of Masson bodies, a histologic feature characteristic of organizing pneumonia (Supplemental Figures 2 and 3 and Supplemental Figure 9E). Notably, there was no evidence in *Scnn1b-*Tg mice for (a) increased numbers of CD4^+^ and CD8^+^ T cells and CD45R^+^ B cells; or (b) differences in iNOS^+^ cells (Supplemental Figure 10A), despite an increase in tertiary lymphoid aggregates (Supplemental Figure 10B).

The long-term (30-day) responses to MA10 infection in *Scnn1b*-Tg mice contrasted with phenotypes reported for aged BALB/c mice, which develop subpleural foci of fibrosis and persistence of KRT8^+^ cells with reduced numbers of *Sftpc^+^* cells within areas of fibrosis (18). Notably, in *Scnn1b*-Tg mice, there was an approximately 2-fold increase in AT2 cell abundance in the chronically diseased regions of *Scnn1b*-Tg mice compared with WT mice at 30 dpi, as measured by pro–surfactant protein C (pro-SPC) IHC (Figure 5, E and F). A small proportion of these AT2 cells colocalized with the marker of proliferation Ki67, suggesting that the alveolar lesion is characterized by increased proliferating cells, some being AT2 cells (Supplemental Figure 11, A and B). Overall, these findings support the characterization of this lesion as an organizing, type 2 inflammatory parenchymal response consistent with pathologic description of chronic eosinophilic pneumonia (CEP).

### Alveolar immune responses to MA10 infection differed already between Scnn1b-Tg and WT mice at 2 dpi.

**Figure 6:**
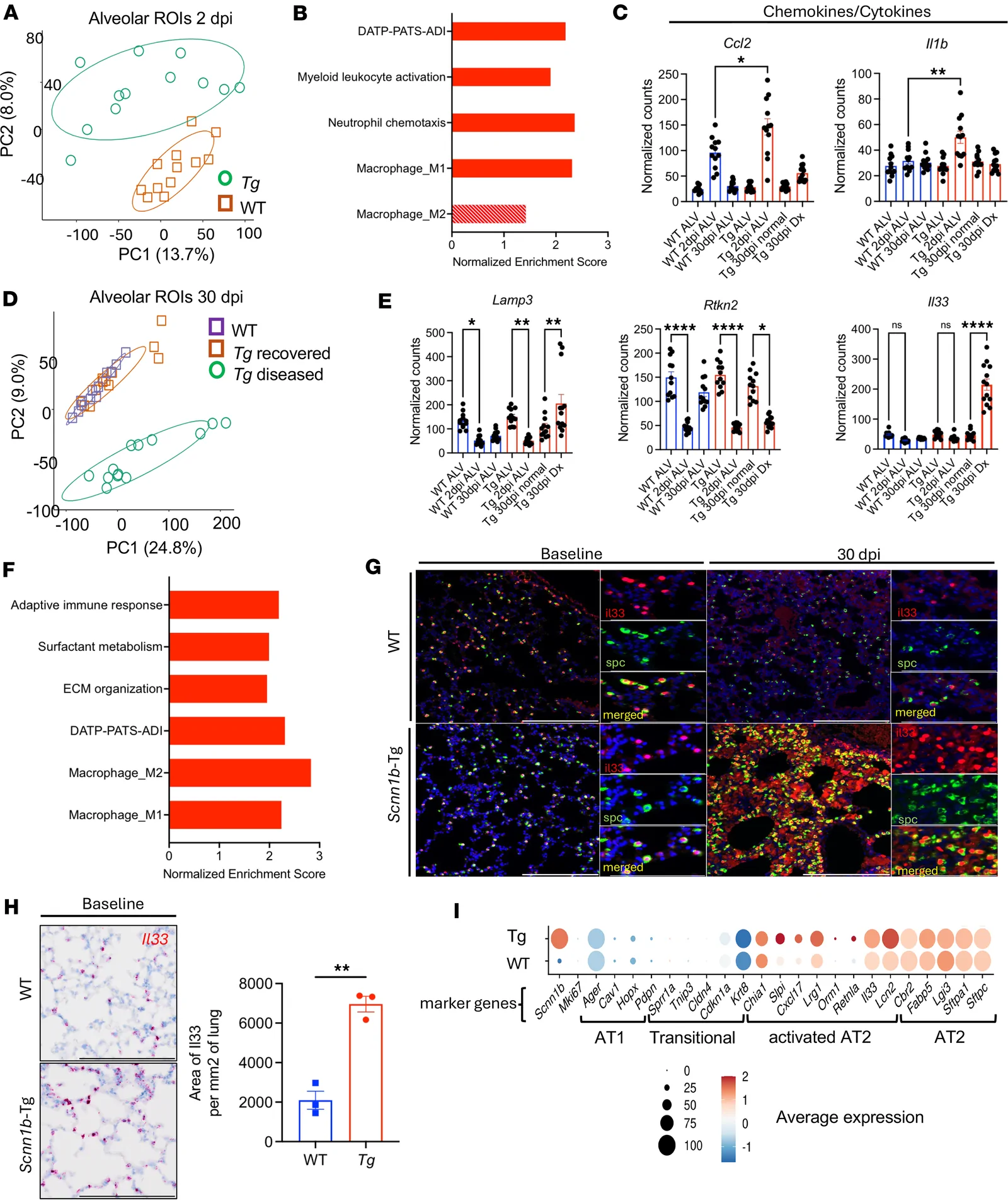
Transcriptional analysis reveals activated AT2 cells and Th2-skewed macrophages at baseline. (**A**) PCA plot of gene expression values from GeoMx spatial transcriptomics of alveolar ROIs from 2 dpi. All ROIs selected are displayed, with green, open circles representing *Scnn1b*-Tg mice and orange, open squares representing WT. (**B**) Normalized enrichment scores and adjusted *P* values for selected gene pathways in alveolar ROIs of *Scnn1b*-Tg mice versus WT mice at 2 dpi. (**C**) Q3-normalized counts representing gene expression of *Ccl2* and *Il1b* across alveolar ROIs at 2 and 30 dpi in WT and *Scnn1b*-Tg mice. For WT mice, all alveoli (ALV) returned to normal by 30 dpi, but in *Scnn1b*-Tg mice, 2 types of alveolar ROIs were selected, those that appeared normal and those that appeared diseased (Dx). (**D**) PCA plot of gene expression from alveolar ROIs from 30 dpi. All ROIs selected are displayed for each experimental group. (**E**) Q3-normalized counts representing gene expression of *Lamp3*, *Rtkn2*, and *Il33* across alveolar ROIs. (**F**) Normalized enrichment scores for selected gene pathways in alveolar ROIs of *Scnn1b*-Tg mice versus WT mice at 30 dpi. (**G**) IHC of IL-33 and pro–surfactant protein C from WT and *Scnn1b*-Tg at 30 dpi and naive mice. DAPI is shown in dark blue. Scale bars: 200 μm and 100 μm (high magnification). (**H**) RNA-ISH of *Il33* in baseline lungs of WT and *Scnn1b*-Tg mice. Quantitation of RNA-ISH *Il33* signal in naive mouse lungs. Scale bar: 200μm. (**I**) Dot plot showing distal lung epithelial markers for WT and *Scnn1b*-Tg mice generated from scRNA-seq data. Dot size indicates percentage expression and dot color average expression. *P < 0.05; **P < 0.01; ****P < 0.0001. ANOVA with Tukey’s multiple-comparison test. Student’s *t* test.

We again used GeoMX DSP to investigate the mechanism by which the *Scnn1b*-Tg alveolar regions were susceptible to the development of CEP after SARS-CoV-2 infection. PCA plots and heatmaps of DEGs among the ROIs selected across genotypes, dpi, and chronic disease regions highlighted distinct differences across all experimental groups (Supplemental Figure 12, A and B, and Supplemental Data File 2). In contrast to proximal airways at 2 dpi (Figure 2C), genotype-specific differences were seen in the alveoli at 2 dpi (Figure 6A). Pathways reflecting increased neutrophil chemoattractants, myeloid leukocyte activation, and M1 macrophage markers were upregulated in *Scnn1b*-Tg compared with WT, reflecting increased expression of inflammatory markers such as *Ccl2* and *Il1b* (Figure 6, B and C, Supplemental Figure 12B, and Supplemental Data File 3). These increased inflammatory signatures occurred despite the reduced expression of viral proteins at 2 dpi in *Scnn1b*-Tg mice (Figure 2B). In parallel, the ISG responses trended toward a reduction in *Scnn1b*-Tg mice (*P* = 0.06; Supplemental Figure 12D), again consistent with lower SARS-CoV-2 Orf1ab transcripts in the alveolar ROIs from *Scnn1b*-Tg mice (Supplemental Figure 12E) and viral IHC quantitation in the alveoli (Figure 2, A and B).

Notably, there were no genotype-specific differences for AT2 (*Sftpa*, *Sftpc*, *Lamp3*), AT1 (*Rtkn2*, *Ager*, *Hopx*), or transitional (*Cdkn1a*, *Krt8*) cell markers in early infection at 2 dpi, with both *Scnn1b*-Tg and WT mice losing AT2 and AT1 cell-type identity markers at 2 dpi and both showing increased transitional cells (Supplemental Figure 12C and Supplemental Data File 1).

Multiplex immunoassays were performed to validate DSP RNA-based inflammatory responses in the alveoli of *Scnn1b*-Tg and WT mice at 2 dpi. The protein level of CCL2, a monocyte chemoattractant (32), in whole-lung homogenates showed a trend (*P* = 0.0519) to be increased at this time point (Supplemental Figure 13B). As predicted from the increase in CCL2 at both the mRNA and protein levels, *Scnn1b*-Tg mice exhibited a greater intrapulmonary influx of Iba1^+^ inflammatory monocytes/interstitial macrophages at 2 dpi than WT mice (Supplemental Figure 13C). Overall, the 2 dpi data suggest that, despite a trend toward reduced viral load in the alveolar space, *Scnn1b*-Tg mice exhibited more robust acute inflammatory responses to viral infection compared with WT mice, which could explain their propensity toward development of the CEP lesions by 30 dpi.

### Late (30 dpi) transcriptional response to MA10 in Scnn1b-Tg alveoli demonstrates a disorganized alveolar architecture.

Consistent with the heterogeneity of alveolar disease observed histologically in *Scnn1b*-Tg at 30 dpi, PCA analysis robustly separated diseased alveoli from histologically normal alveoli in *Scnn1b*-Tg mice at 30 dpi (Figure 6A). Diseased alveolar regions exhibited (a) increases in gene signatures of AT2 cells (*Lamp3*) and alveolar transitional cells (*Krt8*); (b) reductions in AT1 cell signature genes (*Rtkn2*); and (c) activated AT2 cell marker genes, including *Il33* (Figure 6E, Supplemental Figure 12C, and Supplemental Data File 1). Pathway analyses revealed upregulation of pathways associated with surfactant metabolism and transitional cells (damage-associated transient progenitors, pre-alveolar transitional state, alveolar differentiation intermediate [DATP-PATS-ADI]), extracellular matrix (ECM) organization, and adaptive immune response in diseased *Scnn1b*-Tg chronic alveolar lesions (Figure 6F and Supplemental Data File 3). This pattern was juxtaposed to downregulation of blood vessel morphogenesis and smooth muscle contraction (Supplemental Figure 12B). The increased abundance of SFTPC^+^ cells expressing IL-33 was confirmed using immunofluorescence (Figure 6H).

### Predisposition of Scnn1b-Tg mice to CEP: single-cell RNA transcriptional profiling reveals evidence of activated AT2 cells and Th2-skewed macrophages at baseline in Scnn1b-Tg mice.

The presence of CEP in *Scnn1b*-Tg mice but not WT mice suggests that *Scnn1b*-Tg mice exhibit a preexisting susceptibility to this condition. Single-cell RNA sequencing (scRNA-seq) analysis of epithelial cell–enriched whole-lung homogenates from baseline *Scnn1b*-Tg and WT mice was performed to test this hypothesis. A total of 27,169 cells were obtained, and graph-based clustering produced 37 distinct clusters, including epithelial, immune, and stromal cell types (see Methods) (Supplemental Figure 13A). Uniform manifold approximation and projection (UMAP) did not reveal gross separations of cell clusters by genotypes (Supplemental Figure 14, A and B), with both genotypes represented in all cell clusters.

We next specifically analyzed the alveolar cell clusters. A number of mouse scRNA-seq studies have identified an AT2 cell population that emerges early in response to injury, termed “activated AT2 cells,” cells thought to have entered a transitional state that facilitates the generation of AT1 cells (33). Cells that exhibit incomplete transitions from AT2 to AT1 cells, i.e., transitional cells, have been identified in human idiopathic pulmonary fibrosis and postmortem lungs from individuals with COVID-19 (34). AT2 cells from baseline *Scnn1b*-Tg mice exhibited increased expression of marker genes that define activated AT2 cells (e.g., *Il33*, *Lrg1*, *Cxcl17*, *Slpi*) (Figure 6I), suggesting that *Scnn1b*-Tg AT2 cells are activated at baseline. RNA*-*ISH confirmed increased expression of *Il33* in baseline *Scnn1b*-Tg as compared with WT mice (Figure 5F). We speculate that activated AT2 cells mediate the propensity of *Scnn1b*-Tg mice to develop CEP long after MA10 infection.

Our scRNA-seq analyses also revealed differences in alveolar macrophages (AMs) between baseline WT and *Scnn1b*-Tg mice (Supplemental Figure 13C). These changes are consistent with findings from two prior studies describing the transcriptional state of *Scnn1b*-Tg AMs (35, 36). Genes differentiating *Scnn1b*-Tg from WT macrophages included (a) upregulated genes common to both previous reports (*Scd1*, *Igf1*, *Cd63*, *Epas1*); (b) upregulated M2 macrophage genes (*Atf3*, *Cd200*, *Cd36*, *Trem2*); and (c) downregulated M1 macrophage genes (*Cd69*, *Cxcl2*, *Il1b*, *Tlr2*, *Tnf*) (Supplemental Figure 14D). The primed nature of AMs in *Scnn1b*-Tg may also contribute to the CEP phenotype observed in *Scnn1b*-Tg mice.

### Preventative targeting of the IL-33/ST2 axis reduces MA10-induced CEP in Scnn1b-Tg mice.

**Figure 7:**
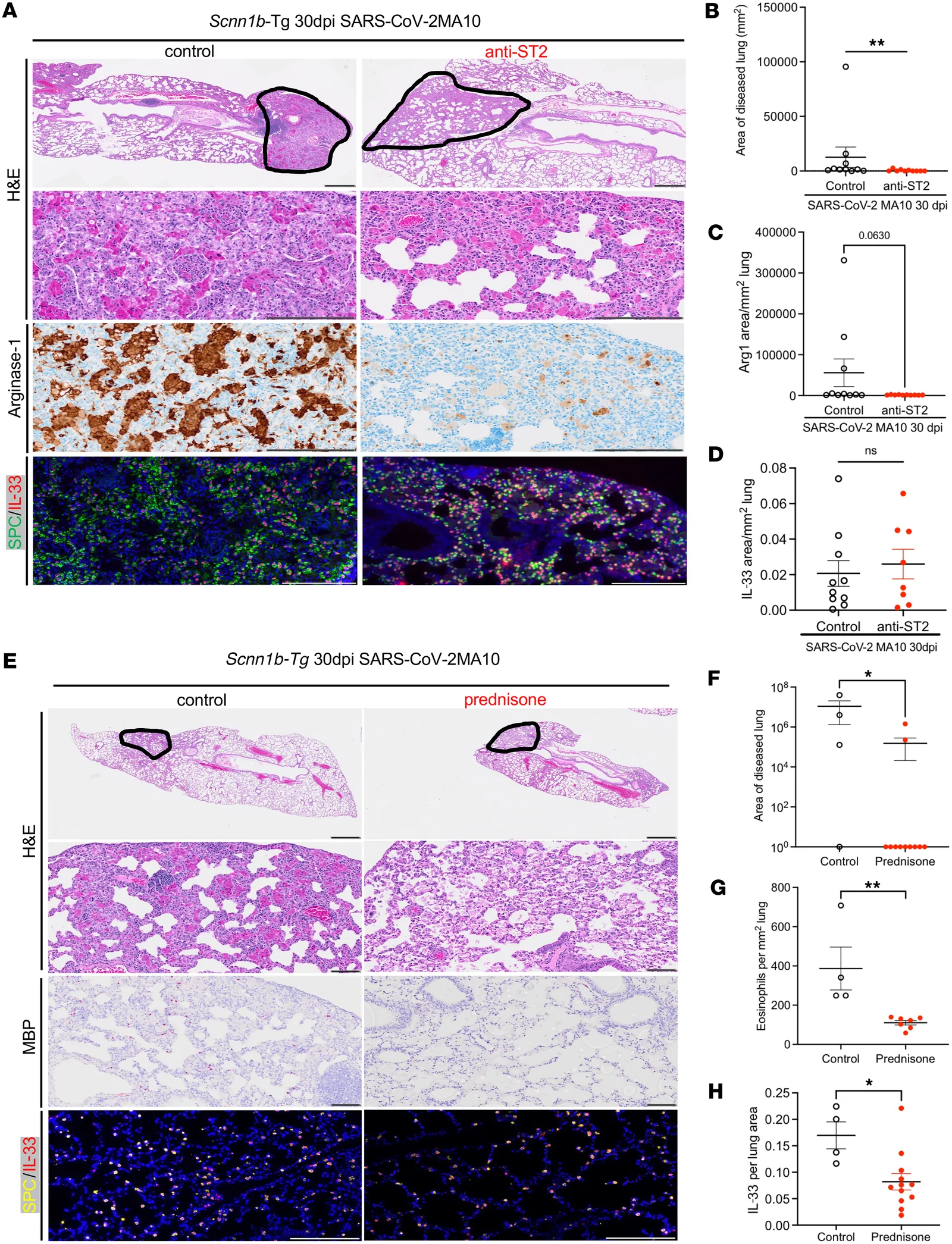
Therapeutic benefit of IL-33 pathway inhibition and prednisone treatment. (**A**) Representative histologic images of lung in *Scnn1b*-Tg mice given vehicle or anti-ST2 monoclonal antibody at 30 dpi. H&E, hematoxylin and eosin. Arginase-1 indicates DAB-labeling (brown color) IHC for arginase-1. SPC/IL-33 indicates IHC (fluorescent) for pro–surfactant protein C and IL-33. Scale bars: 1 mm (low magnification) and 200 μm. (**B**) Quantitation of the area of diseased lung per total lung area. (**C**) Quantitation of arginase-1 IHC–positive area per total lung area. (**D**) Quantitation of IL-33^+^ area per lung area. (**E**) Representative histologic images of lung in *Scnn1b*-Tg mice treated with vehicle or oral prednisone at 30 dpi. H&E, hematoxylin and eosin. MBP indicates IHC (red) for major basic protein. SPC/IL-33 indicates IHC (fluorescent) for pro–surfactant protein C and IL-33. Scale bars: 1 mm (low magnification) and 100 μm. (**F**) Quantitation of the area of eosinophilic pneumonia per total lung area. (**G**) Quantitation of arginase-1 IHC–positive area per total lung area. (**H**) Quantitation of IL-33^+^ area per lung area. *P < 0.05; **P < 0.01. Student’s *t* test.

Based on our finding of upregulation of IL-33 in baseline and post-MA10-infected *Scnn1b*-Tg diseased lungs (Figure 6, G and H), a monoclonal antibody against the canonical IL-33 receptor (ST2) was tested for efficacy in a pretreatment protocol (Supplemental Figure 15A). The anti-ST2 monoclonal antibody pretreatment protocol had no impact on weight loss, viral titer, or an array of cytokines in either genotype at 2 dpi (Supplemental Figure 15, B–D). However, infected *Scnn1b*-Tg mice given anti-ST2 monoclonal antibodies exhibited a reduction in the magnitude of CEP lesions as compared with vehicle-treated *Scnn1b*-Tg mice (Figure 7, A and B). A trend toward reduced accumulations of alternatively activated macrophages but not eosinophils was noted (Figure 7C and Supplemental Figure 15E). Hyperplasia of IL-33^+^ AT2 cells with upregulated IL-33 expression persisted in the presence of the anti-ST2 monoclonal antibody (Figure 7, A and D).

### Post-infection corticosteroid administration is also effective in treating MA10-induced Scnn1b-Tg eosinophilic pneumonia.

*Scnn1b*-Tg and WT mice infected with MA10 were fed enteral prednisone (37) or vehicle control, commencing at 7 dpi (Supplemental Figure 16A). Serum prednisone concentrations were confirmed by ultra-high-performance liquid chromatography/mass spectrometry to be within a range that has previously been reported in mice (Supplemental Figure 16B) (37). *Scnn1b*-Tg mice treated with control diet exhibited greater weight gain from 9 dpi to 30 dpi than *Scnn1b*-Tg mice that received prednisone diet (Supplemental Figure 16C). Compared with vehicle treatment, prednisone reduced the area of diseased lung in *Scnn1b*-Tg mice more than 10-fold (Figure 7, E and F), significantly reduced the abundance of eosinophils (Figure 7G), and reduced expression of IL-33 in AT2 cells (Figure 7H and Supplemental Figure 16D). The reduction in lesion area was also accompanied by fewer lesions that were positive for arginase-1 (Supplemental Figure 16D).

## Discussion

Although the evidence for the burden of PASC (ongoing, relapsing, or new symptoms or conditions present 30 or more days after infection) is compelling (13, 17), data describing the long-term sequelae of COVID-19 in individuals with preexisting chronic lung disease remain limited. Except for post-COVID pulmonary fibrosis, few mechanistic studies of the spectrum of reported PASC pulmonary endotypes and endotype-specific countermeasures are available (18). Accordingly, we focused our studies on understanding the PASC pulmonary phenotypes in the *Scnn1b*-Tg model of chronic bronchitis.

*Scnn1b*-Tg mice exhibited a less severe acute MA10 pulmonary infection than WT mice (Figure 1). An intact mucus layer has been shown in vitro to reduce SARS-CoV-2 infection of human bronchial epithelial cell cultures (38). Our data imply that resistance to viral entry and/or replication in the *Scnn1b*-Tg mouse includes not only the barrier effect of hyper-concentrated airway mucus, but also an epithelium primed for antiviral responses in concert with the reported activity of an increased number of innate immune cells on and within airway surfaces (Figure 2) (39).

Despite less severe acute illness, a fraction of *Scnn1b*-Tg mice developed severe, focal alveolar and airway disease by 30 dpi (Figures 1 and 3). Histology studies of MA10-infected *Scnn1b*-Tg mice at 30 dpi identified parenchymal lesions characterized by increased eosinophil numbers, M2-skewed macrophage accumulation, and AT2 hyperplasia, all characteristic of chronic eosinophilic pneumonia (CEP). With respect to the pathogenesis of this response, AT2 cells from baseline *Scnn1b*-Tg mice exhibited an activated (IL-33–high) basal state as evidenced by scRNA-seq data and confirmed by RNA-ISH (Figure 5). This activated AT2 cell state, also known as a pre-alveolar transitional state (PATS), has previously been identified as early-onset response following acute lung injury (33). We speculate that the basally IL-33–activated AT2 cells responded to MA10 infection with T2-skewed hyperinflammatory responses to MA10 infection in *Scnn1b*-Tg mice at 2 dpi. Notably, our longitudinal transcriptional data indicated that the baseline IL-33–high activated basal AT2 cell state was amplified by MA10 infection at 30 dpi in *Scnn1b*-Tg mice, a finding confirmed by increases in IL-33 protein via immunofluorescence. Moreover, transcriptional data demonstrated that an *Il33*-expressing PATS transitional cell signature persisted in *Scnn1b*-Tg MA10-infected mice in concert with an increased AT2 cell abundance. Notably, in contrast to studies of MA10-induced pulmonary fibrotic responses in BALB/c mice (18), the alveolar regions in *Scnn1b*-Tg mice at 30 dpi were populated by increased numbers of AT2 cells with little evidence of diffuse fibrosis.

Our transcriptional data also shed light on the mechanism that stimulated the M2 macrophage/eosinophil migration into alveolar spaces to produce a CEP-like pathology. Positive feedback cycles exist between AT2 cells and pulmonary macrophages. For example, the macrophage/monocyte chemokine Ccl2, released by AT2 cells, is a key component for (a) monocyte recruitment; and (b) propagation of signals from macrophages, e.g., via IL-1β and other cytokines, to transitional AT2 cells to produce AT2 cell hyperplasia (40). Additionally, it has been shown that IL-33 is capable of recruiting monocyte-derived macrophages after influenza A virus infection (41). Our data demonstrate that *Scnn1b*-Tg mice exhibit both baseline evidence of macrophage activation and at 2 dpi an increased expression of monocyte chemokines (Ccl2) and activated macrophage products (IL-1α). We speculate that the robust alveolar inflammatory cell response of *Scnn1b*-Tg mice to MA10 infection at 2 dpi amplified the T2-skewed AT2 cell activation/transitional cell states that produced CEP in the 30 dpi interval.

Airway lesions characterized by eosinophilia and mucus metaplasia, with evidence of increased basal cell marker genes, were typically observed in the approximately 40% of *Scnn1b*-Tg mice that exhibited alveolar disease, suggesting a common T2 pathophysiology (Figure 3). This airway finding has been described in human in vitro studies, in which basal cells from SARS-CoV-2–infected individuals exhibit a T2 inflammatory phenotype and undergo goblet cell hyperplasia, with a relative increase in MUC5AC versus MUC5B (42). Increased expression of *MUC5AC* has been demonstrated by others using scRNA-seq of bronchial brushings from individuals with PASC (7).

A candidate upstream cytokine that triggers CEP-like and T2-skewed airway sequelae in *Scnn1b*-Tg mice after MA10 infection is IL-33. IL-33 is an epithelial cell–derived cytokine released following cell injury or cell death and initiates type 2 inflammatory responses (43). IL-33 acts predominantly via a canonical receptor, i.e., the serum stimulation-2 (ST2) receptor. IL-33 is elevated in bronchoalveolar lavage from naive, juvenile *Scnn1b*-Tg mice, which, based on our data, likely reflects AT2 cells at the source (44). *Il33* knockout in *Scnn1b*-Tg mice also reduced levels of airway T2-associated genes, bronchoalveolar lavage fluid eosinophils, and MUC5AC protein expression in airway epithelia (44). There was no identified impact of the anti-ST2 monoclonal antibody on the severity of acute disease, consistent with findings from clinical trials in humans with severe COVID-19 (45). However, the role of IL-33, via ST2 signaling, in the development of the *Scnn1b*-Tg CEP endotype was supported by data from the anti-ST2 monoclonal antibody administration protocols (Figure 6). The CEP-diseased areas of the *Scnn1b*-Tg lung were reduced more than 10-fold, but the eosinophilia, alternatively activated macrophage accumulations, and AT2 cell hyperplasia were not completely blocked by the ST2 monoclonal antibody. Note that recent evidence has shown that IL-33 acts also via the receptor for advanced glycation end products (RAGE) to signal by way of epidermal growth factor to promote epithelial mucin hypersecretion. Future studies to explore this pathway, which is distinct from IL-33/ST2 signaling (46), are warranted.

The *Scnn1b*-Tg CEP response to MA10 mimics pathology from case reports of CEP in humans after SARS-CoV-2 infection (14, 16, 47). However, the incidence of this PASC response to SARS-CoV-2 in humans is difficult to predict, because neither CT scans nor blood eosinophils are sufficient for a CEP diagnosis. Indeed, a bronchoscopy demonstrating bronchoalveolar lavage eosinophilia or transbronchial biopsy is required for diagnosis. Human cases of CEP and cryptogenic organizing pneumonia are typically responsive to corticosteroids. Like human CEP, prednisone started at 7 dpi attenuated CEP in *Scnn1b*-Tg mice with about 10-fold reductions in diseased areas and eosinophil abundance (Figure 6). The observation that prednisone reduced IL-33 expression in AT2 cells may suggest that a component of prednisone’s therapeutic benefit in this study was manifest by inhibition of an IL-33–dependent mechanism.

There are some limitations to our study. First, all long-term experiments were carried out exclusively in female mice owing to housing constraints within our biosafety level 3 (BSL3) facility. With respect to the human population, females have a propensity to develop chronic disease phenotypes following SARS-CoV-2 infection (48), and the incidence of CEP is greater in the female population (49). It will be important to investigate in the future whether the incidence of CEP is similarly increased in female as compared with male *Scnn1b*-Tg mice after SARS-CoV-2 infection and whether responses to corticosteroids and/or to ST2 antibodies differ by sex. Second, all animal inoculations were carried out with the MA10 virus, developed from the USA-WA1 strain. Although recent work has indicated generally similar but milder phenotypes with subsequent SARS-2 variants (50), without experimental evidence we cannot be certain that the MA10 data can generalize to all SARS-2 respiratory viruses. Third, we did not investigate whether this disease phenotype was specific to SARS-CoV-2, and it would be informative to explore whether this disease phenotype occurs with other respiratory viruses. Fourth, with respect to the natural history of the CEP lesions, our studies were largely limited to a maximum of 60 dpi, and it remains unclear whether CEP naturally resolves over longer time periods. Limitations imposed by the BSL3 facility also prevented flow cytometry studies and bronchoalveolar lavage collections that would be required to investigate the interactions between IL-33–secreting cells and IL-33–dependent effector cells, e.g., ILC2 and T2 lymphocytes, that may mediate CEP pathogenesis. Finally, constitutive expression of IL-33 differs between mice and humans. Epithelial expression is dominated by AT2 cells in mice versus airway basal cells in humans (43).

In summary, the *Scnn1b*-Tg model of chronic bronchitis exhibits an IL-33–driven chronic PASC response that resembles case reports of CEP in humans post–SARS-CoV-2 infection. We speculate that a subgroup of human subjects who express a phenotype consistent with COPD-like airways and alveolar disease may have an increased incidence of CEP after SARS infection and exhibit benefit with anti–IL-33 antibody therapy.

## Methods

### Sex as a biological variable.

Because of the animal housing limitations within the BSL3 facility, only female mice were studied. Sex differences have been demonstrated in response to SARS-CoV-2 in mice. As we only studied female mice, results must be interpreted in this context.

### In vivo infection.

Ten- to twelve-week-old congenic C57BL/6N *Scnn1b*-Tg and WT littermates were bred and genotyped at the University of North Carolina at Chapel Hill as previously described (51). One week before inoculation, mice were transferred into the BSL3 laboratory for acclimation. At day 0, mice were anesthetized with a mixture of ketamine and xylazine followed by inoculation with indicated PFU of mouse-adapted SARS-CoV-2 MA10 via the intranasal route (19). Mice were monitored daily for changes in body weight and signs of morbidity. At indicated time points, mice were sacrificed via inhalation anesthesia (4%–5% isoflurane) followed by exsanguination and collection of vital organs. Lung lobes were collected as follows: Inferior right lobes were collected in vials containing 1 mL PBS and homogenized with glass beads for viral titer determination via plaque assay and analyses of chemokines/cytokines via Bio-Plex; and left lung lobes were immersion-fixed in 10% formalin for histopathologic evaluation.

### Plaque assay.

Inferior right lung lobes were used for determination of viral loads as previously described (19). Briefly, tissue samples were homogenized in PBS, serially diluted, and used to infect monolayers of Vero E6 cells, followed by agarose overlay. After 72 hours of incubation, plaques were visualized via Neutral Red Dye, counted, and used to calculate the total number of replication-competent viral particles per sample.

### Histologic analysis and staining.

Left lung lobes (or whole lungs) were collected on indicated days and immersion-fixed in 10% phosphate-buffered formalin to avoid dislodgment of luminal contents. After storage for at least 7 days at 4°C under BSL3 containment, samples were processed, embedded in paraffin, and sectioned at 5 μm thickness as previously described (18) for histochemical (hematoxylin and eosin [H&E] for general morphology, AB-PAS for glycoconjugates, Picrosirius red for collagen deposition) or IHC stain.

### Immunohistochemistry.

Immunohistochemistry (IHC) was performed using either the automated Leica Bond III Autostainer system or manual stains (18, 52, 53). In brief, sections were incubated at 60°C for at least 2 hours, deparaffinized with xylene for a total of 10 minutes, and rehydrated with graded ethanol exposures (100%, 95%, and 70% ethanol for 5 minutes each). For stains using peroxidase-based detection, i.e., DAB substrate, quenching of endogenous peroxidase was performed with hydrogen peroxide (0.5%) in methanol for 15 minutes. Antigen retrieval was performed in 0.1 M sodium citrate, pH 6.0, using a steamer (37530A, Hamilton Beach) for 20 minutes. After cooling and rinsing with distilled water, the slides were washed with PBS, and blocking was performed with Blocking One buffer (03953-95, Nacalai Tesque) at room temperature for 30 minutes. The following primary antibodies were applied to the slides and incubated at 4°C overnight in the dark: rabbit anti–α-smooth muscle actin antibody (ab124964, Abcam; 1:2,000), rabbit anti–arginase-1 (93668S, Cell Signaling Technologies; 1:100), rabbit anti-CD4 (ab183685, Abcam; 1:1,000), rat anti-CD8 (4SM15, eBiosciences; 1:100), rat anti-CD45R (550286, BD Pharmingen; 1:500), rabbit anti-EPX (PA5-62200, Invitrogen; 1:1,000), rat anti-MBP (Mayo Clinic, Scottsdale, Arizona), goat MPO (AF3667, R&D; 1:200), goat anti–IL-33 (AF3626, R&D; 1:250), rabbit anti–pro-SFTPC (ab90716, Abcam; 1:100), rabbit anti-MUC5B (H-300, Santa Cruz Biotechnology; 1:1,000), and rabbit anti–SARS-CoV-2 nucleocapsid protein (1087200, Novus Biologicals; 1:500). Sections were washed with PBS plus 0.1% Tween 20 (PBS-T) and incubated with secondary antibody at room temperature for 1 hour. For DAB staining, the slides were treated with Vectastain Elite ABC Kit (PK-6100, Vector Laboratories) followed by visualization with DAB and counterstaining with fast red. For fluorescent staining, Vector TrueVIEW Autofluorescence Quenching Kits (SP-8400, Vector Laboratories) were used to reduce autofluorescence. The slides were mounted with DAPI-containing mounting medium (H1800, Vector Laboratories) to visualize nuclei. Stained mouse tissue sections were scanned and digitized using an Olympus VS200 slide scanner.

### RNA in situ hybridization.

RNA in situ hybridization (RNA-ISH) was performed on FFPE mouse and human lung tissues as described previously (21). RNAscope 2.5 HD Reagent Kit–RED (322350, ACD), RNAscope 2.5 HD Duplex Reagent Kit (322430, ACD), BaseScope Detection Reagents v2–RED (323910, ACD), and RNAscope Multiplex Fluorescent Reagent Kit v2 (323100, ACD) were used according to the manufacturer’s instructions. Tissue sections were deparaffinized with xylene (twice for 5 minutes) and 100% ethanol (twice for 1 minute) and incubated with hydrogen peroxide for 10 minutes, followed by target retrieval in boiling water for 15 minutes and incubation with Protease Plus (322330, ACD) at 40°C for 15 minutes. Slides were hybridized with custom probes at 40°C for 2 hours using a HybEZ Oven (241000, ACD) and signals amplified according to the manufacturer’s instructions. After counterstaining with hematoxylin or DAPI, stained slides were scanned and digitized using an Olympus VS200 light or fluorescent microscope with a ×20 0.80 NA, ×40 0.95 NA, or ×60 1.42 NA objective (21). Probes for ubiquitin C (*UBC*) and bacterial *DapB* genes were used for positive and negative controls, respectively.

### In vivo ST2 monoclonal antibody administration.

For monoclonal antibody studies, mice were treated intraperitoneally with 500 μg/mouse ST2 monoclonal antibody 2–3 hours before and on days 2, 4, 7, 11, 14, 16, 18, 21, 23, 25, and 28 after infection. Treated and infected mice were monitored for weight loss and mortality and sacrificed on day 30 after infection. The anti-ST2 monoclonal antibody was initially developed by Amgen Inc. and was obtained from Genentech Inc.

### Administration of oral prednisone to mice.

Prednisone was administered in the diet. Control diet was based on the purified, powdered diet defined by American Institute of Nutrition–93G guidelines. Prednisone diet was prepared as previously described (37) to yield an approximately 50 mg/kg daily dose of prednisone over the duration of the treatment. The selected dose equates to a human equivalent dose of 46 mg/d. Prednisone content was verified by liquid chromatography/tandem mass spectrometry. Diets were stored at –20°C and provided to mice ad libitum in feeding jars.

### Prednisone pharmacologic analyses.

Mice were dosed as above. Serum was collected at harvest and analyzed using ultra-high-performance liquid chromatography/time-of-flight mass spectrometry (UHPLC–TOF MS). Samples were prepared by dilution 1:1 with water, then precipitation of protein 3:1 with acetonitrile (Sigma-Aldrich) containing ^13^C_3_ prednisone (Cerilliant) as an internal standard. Samples were allowed to stand at room temperature for 1 hour, then centrifuged. The supernatant was separated using a Flexar FX-20 UHPLC system (PerkinElmer) with a Kinetex C18 biphenyl column (2.6 μm, 50 mm by 3 mm; Phenomenex) at 27°C with 100% MS-grade water (Sigma-Aldrich), 0.1% formic acid (Thermo Fisher Scientific), and 100% acetonitrile (Supelco) and 0.1% formic acid (Thermo Fisher Scientific) linear 2.5-minute gradient elution at a flow rate of 0.6 ml/min. The PerkinElmer Axion2 TOF mass spectrometer operated in positive-ion electrospray ionization mode was used to detect accurate mass spectra of prednisone at 359.1853 [M + H]^+^. The method was linear from 1 to 20 ng/mL with a lower limit of detection of 1 ng/ml.

### Bio-Plex immunoassay.

Chemokine and cytokine analyses were performed on clarified lung homogenates using the Bio-Plex Pro mouse cytokine 23-plex assay (Bio-Rad Laboratories) according to the manufacturer’s instructions. Briefly, 50 μL of lung homogenate was incubated with magnetic beads and, after several wash steps, treated with detection antibody and streptavidin-PE. A MAGPIX machine (Luminex) and xPONENT software were used to record results, and individual chemokines and cytokines were quantitated via comparison with a standard curve.

### Preparation of lung cell suspensions for scRNA-seq analysis.

After sacrifice, the lungs were perfused with cold saline using a 25-gauge needle and a 10 mL syringe via the right ventricle to clear the lung of circulating blood. The lungs were inflated through the trachea with 1 mL of digestion mix, consisting of collagenase type I (450 U/mL; catalog 17100-017, Gibco), Dispase (5 U/mL; catalog 354235, BD Biosciences), and DNase I (1 mg/mL; catalog 10104159001, Roche) in DMEM/F12. Lungs were dissected out of the thoracic cavity and extraneous tissues removed. Lungs were minced manually with scissors in a 1.5 mL Eppendorf tube and then incubated in 4 mL of digestion mix for 25 minutes at 37°C with constant agitation. The homogenate was pipetted with a 1,000 μL wide-orifice pipette and then centrifuged at 400*g* (1,500 rpm) for 7 minutes. The supernatant was removed, and the pellet was resuspended in 5 mL Accutase with 5 mM EDTA for 10 minutes at 37°C with constant agitation. This was then filtered using a 70 μm filter, followed by centrifugation of the flowthrough at 400*g* for 5–8 minutes at 4°C. The supernatant was removed, and 2 mL of red blood cell lysis buffer was added for 2 minutes at room temperature. Five milliliters of DMEM/F12 plus 10% FBS was added to stop red blood cell lysis. Further centrifugation was then performed at 400*g* for 5–8 minutes at 4°C. The pellet was resuspended in 1 mL of FACS buffer (PBS, 1.5% BSA, 2 mM EDTA) and filtered through a 40 μm filter (H13680-0040, Bel-Art). Cells were counted and viability checked using a hemocytometer by trypan blue exclusion. The concentration was adjusted to 1 × 10^7^ cells in 100 μL of FACS buffer for flow cytometry staining and sorting.

### Flow cytometry sorting of lung cell suspensions.

For each sample, Fc receptor blockade was performed with rat anti–mouse FcγRIII/II receptor (CD16/32; BD Biosciences) for 5 minutes on ice. Cells were stained using CD45 (BD Biosciences), CD326 (BD Biosciences), and viability stain (Zombie NIR, BD Biosciences) for 1 hour, as previously described (54). A BD FACSAria II flow cytometry cell sorter was used for cell sorting. Dead cells were excluded, and cells were sorted into CD326^+^CD45^–^ (epithelial), CD326^–^CD45^+^ (immune), and CD326^–^CD45^–^ (non-immune, non-epithelial). These cells were then combined in a 2:2:1 ratio to enrich for epithelial cell populations (CD326^+^) and deplete the immune cell population (CD45^+^).

### Chromium 10x Genomics single-cell RNA-seq library preparation and sequencing.

A 10x Chromium device was used for single-cell capture, as described previously (55). Cell suspensions were loaded on a Chromium controller instrument (10x Genomics) to generate single-cell Gel Beads-in-emulsions, as previously described. Libraries were prepared according to the Single Cell 3′ v2 Reagent Kits user guide (10x Genomics). Sequencing libraries were loaded on a NovaSeq 6000 SP (Illumina) with a custom sequence setting of 28 × 8 × 91, to obtain a sequencing depth of at least 5 × 10^4^ reads per cell.

### Single-cell RNA-seq primary data analysis.

Raw data in FASTQ format were mapped to current mouse genome reference (GRCm39) and gene annotation (GENCODE mouse vM26) using Cell Ranger tools v6.0.1 (10x Genomics) with default parameters (include introns = false). The resultant gene × cell barcode matrices from individual samples were processed, merged, and integrated in R following recommended workflows from Seurat (56). Briefly, preprocessing and quality control at the individual sample level were performed using SoupX for ambient RNA decontamination, doublet removal was performed with scDblFinder, and the resultant matrix was saved into a Seurat dataset. Cell barcodes were filtered for minimal gene count >400, maximal gene count between 5,000 and 6,000, and percentage mitochondrial gene expression <10%, before multiple samples were merged and integrated using about 4,000 variable genes (IntegrateData function from Stuart et al., ref. 56). Cell clusters from integrated data were derived from nearest-neighbor graphs using 40 PCA dimensions and cluster resolution of 0.5 following Seurat recommendations. Differential gene expression analysis was performed using the hurdle method and the Bioconductor R package MAST (57), which combines testing of proportion of positive cells and of mean gene expression levels among positive cells between treatment groups. Gene set enrichment analysis (GSEA) based on log fold change ranks was performed using the Bioconductor R package fgsea (58) against gene set collections obtained from Gene Ontology biological process (59) and Reactome pathways (https://reactome.org/). Custom pathways and gene lists of interest were curated manually from published literature and appended to the Reactome pathways. Various plots were produced using R libraries such as Seurat, ggplot2 (60), plotly (https://plotly-r.com), and ComplexHeatmap (61).

### GeoMx digital spatial profiling.

Five-micrometer-thick FFPE sections were prepared using the NanoString Technologies protocol as previously described (12). Before imaging, nuclei were visualized with 500 nM Syto83 (Invitrogen). Mouse tissue morphology was visualized by H&E, and RNA-ISH for SARS-CoV-2 spike mRNA. Mouse Whole Transcriptome Atlas probes with COVID-19 spike-in gene targets (spike and Orf1ab) were hybridized, after which slides were washed twice in 2× SSC and loaded on the GeoMx Digital Spatial Profiler (DSP) (Nanostring). Regions of interest (ROIs) were selected per section from scanned ×20 images of complete lung sections, either H&E or RNA-ISH of SARS-CoV-2 spike mRNA. ROI selection was made as outlined in Supplemental Figure 3A. The GeoMx device exposed the selected ROIs to 385 nm light (UV), releasing the indexing oligonucleotides and collecting them via microcapillary into distinct wells. Indexing oligonucleotides were deposited in a 96-well plate, dried overnight, and resuspended in 10 μL of DEPC-treated water. Oligonucleotides from each ROI were indexed using unique i5 and i7 dual-indexing systems. Four microliters of indexing oligonucleotides were used in each PCR reaction. PCR reactions were purified twice using AMPure XP beads (A63881, Beckman Coulter) according to the manufacturer’s protocol. Libraries were paired-end-sequenced (2 × 75) on a NextSeq 2000 (Illumina), which provided up to 800 million paired-end reads total aligned reads.

### GeoMx digital spatial profiling data analysis.

Raw count and upper quartile (Q3)–normalized count data were exported from the NanoString local DSP server. Normalized Q3 counts were used for principal component analysis (PCA). PCA was performed using the R package ade4 (62) and visualized using the factoextra and ggplot2 packages. Differential gene expression analyses from raw count data were performed using the dream function from the Bioconductor R package variancePartition (63) to account for related ROIs from the same lung and assay slides as random-effect factors, and PC1 as a covariate to adjust for sample quality. Pre-ranked GSEA was performed using the Bioconductor R package fgsea (58). Hierarchical clustering heatmaps were generated using the Bioconductor R package ComplexHeatmap.

### Mouse lung morphometric analysis and quantitation.

Stained sections were scanned and digitized using an Olympus VS200 microscope with a ×40 1.35 NA objective. Images were imported into Visiopharm Software (version 2020.09.0.8195) for quantitation. Lung tissue, IHC, and RNA-ISH signal were quantified using customized analysis protocols to classify (a) lung tissue ROI using a decision forest classifier, and (b) area of positive probe signal based on the thresholded signal, i.e., excluding background, in the channel corresponding to the relevant probe. For DAB-based probes, thresholds were determined using a contrast of red-blue channels. For fluorescent probes, either immunofluorescence or fluorescent multiplex RNA-ISH, thresholds were determined using FITC/TRITC/Cy3/Cy5/Cy7 signal corresponding to specific immunofluorescence and RNA-ISH gene probes. Test and control slides were analyzed under the same conditions. Results were expressed as probe-positive area relative to total lung tissue area, unless otherwise stated.

### Statistics.

Statistical analyses were performed using GraphPad Prism version 10. Statistical significance was evaluated using unpaired, 2-tailed Student’s *t* test or 1-way ANOVA (multiple comparisons) with Dunnett’s test or 2-way ANOVA (multiple comparisons) with Tukey’s test. Data are presented as mean ± SEM. *P* < 0.05 was considered statistically significant.

### Study approval.

Mouse studies were performed at the University of North Carolina (Animal Welfare Assurance A3410-01) using protocols approved by the University of North Carolina at Chapel Hill (UNC) Institutional Animal Care and Use Committee.

### Data availability.

All data relevant to the conclusions of this paper are present in the paper or the supplemental material. Additional DSP data are available in the Supplemental Data Files. Single-cell RNA-seq data were deposited in the NCBI’s Gene Expression Omnibus database (GEO GSE310468). A Supporting Data Values file of all data points shown in this article is available as supplemental material.

## Author contributions

PEH, WKO, ALB, RSB, RJP, and RCB designed research studies. PEH, SRL, JBH, KO, and AS conducted experiments. RCG, LCM, MS, and EFB acquired data. JRH, HD, EFB, SAS, KO, and MS analyzed data. JJP, LKH, and JRR provided reagents. PEH, SRL, RJP, RSH, ALB, RS, WKO, and RCB wrote the manuscript. All authors read and approved the final manuscript.

## Conflict of interest

JRR is a current employee of Genentech Inc. WKO and RCB are listed on the patent for the *Scnn1b*-Tg mice used in this study, US Patent 7,772,458 B2.

## Funding support

This work is the result of NIH funding, in whole or in part, and is subject to the NIH Public Access Policy. Through acceptance of this federal funding, the NIH has been given a right to make the work publicly available in PubMed Central.

- NIH P30 DK065988 (to RCB) and U19 AI116484 (to RSB).
- Rapidly Emerging Antiviral Drug Development Initiative (READDI) at the University of North Carolina at Chapel Hill, appropriated by the North Carolina General Assembly (RCB and RSB).
- Cystic Fibrosis Foundation ESTHER24R0 (to RCB), HAWKIN21F0 (to PEH), and 00167G220 (to RJP).

## Supplementary Material

Supplemental data

Supplemental data set 1

Supplemental data set 2

Supplemental data set 3

Supporting data values

## Version 1 07/07/2026

In-Press Preview

## Version 2 08/24/2026

Electronic publication
