# Supplementary material for: SARS-CoV-2 infection produces an IL-33–dependent chronic eosinophilic pneumonia and muco-inflammatory airways disease in Scnn1b-Tg mice
Source: JCI Insight. 2026 Jul 7;11(16):e203283. doi: 10.1172/jci.insight.203283 (PMC13502183; doi:10.1172/jci.insight.203283)
Supplement: Supplementary file 1 [file jciinsight-11-203283-s305.pdf]

Figure S1

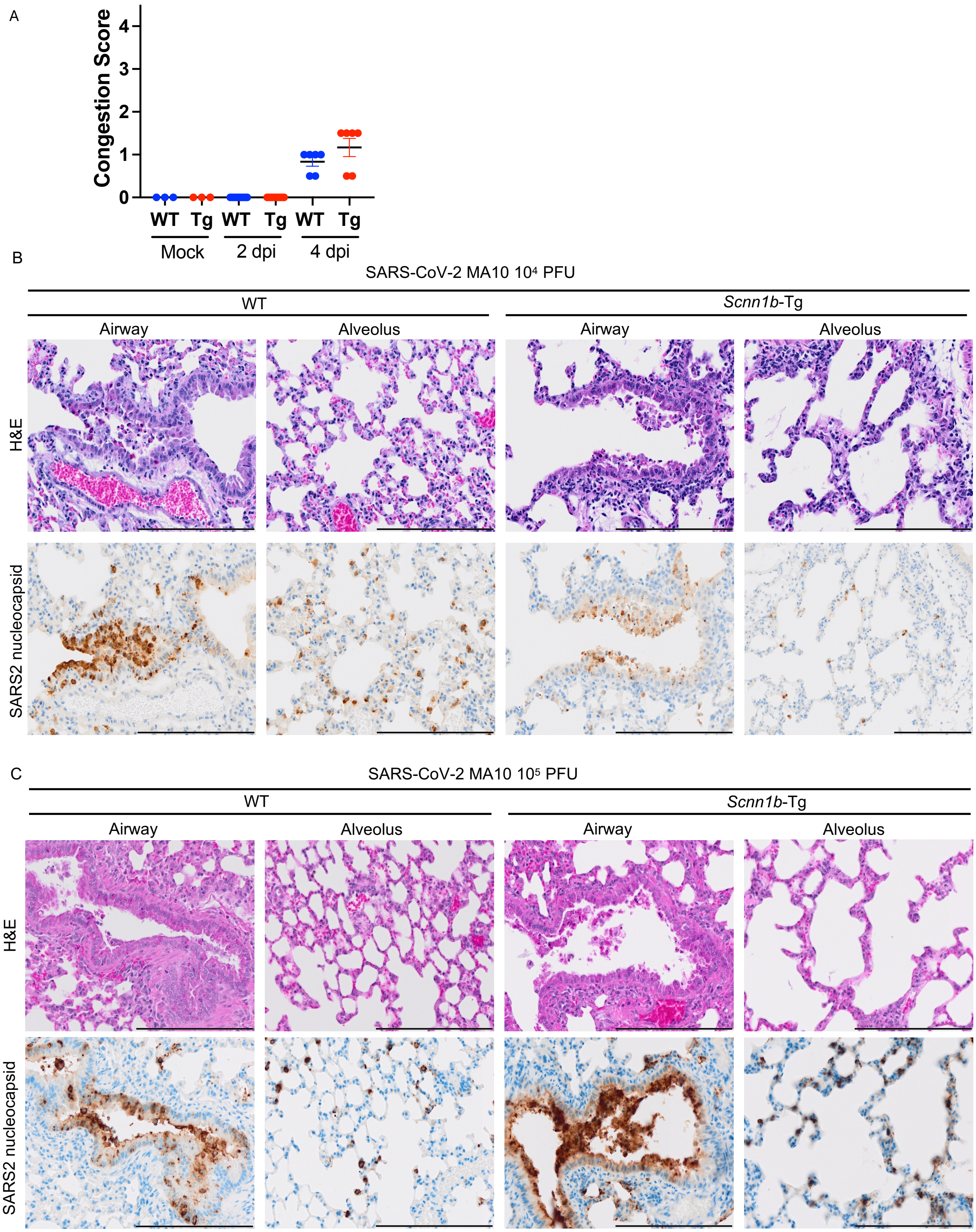

Figure S1: *Scnn1b*-Tg mice are protected from acute sequelae of SARS-CoV-2 and later develop chronic lung disease. (A) Congestion score for 12-week-old WT and *Scnn1b*-Tg mice infected with 10^4 PFU of SARS-CoV-2 MA10 virus ($p>0.05$ between WT and *Scnn1b*-Tg for all conditions). (B) Histopathological analysis of lungs at 2 dpi with 10^4 PFU of SARS-CoV-2 MA10. H&E indicates hematoxylin and eosin. SARS2 nucleocapsid indicates DAB-labeling (brown color) IHC for the nucleocapsid protein of SARS-CoV-2. (C) Histopathological analysis of lungs at 2 dpi with 10^5 PFU of SARS-CoV-2 MA10. Scale bars represents 200μm.

Figure S2

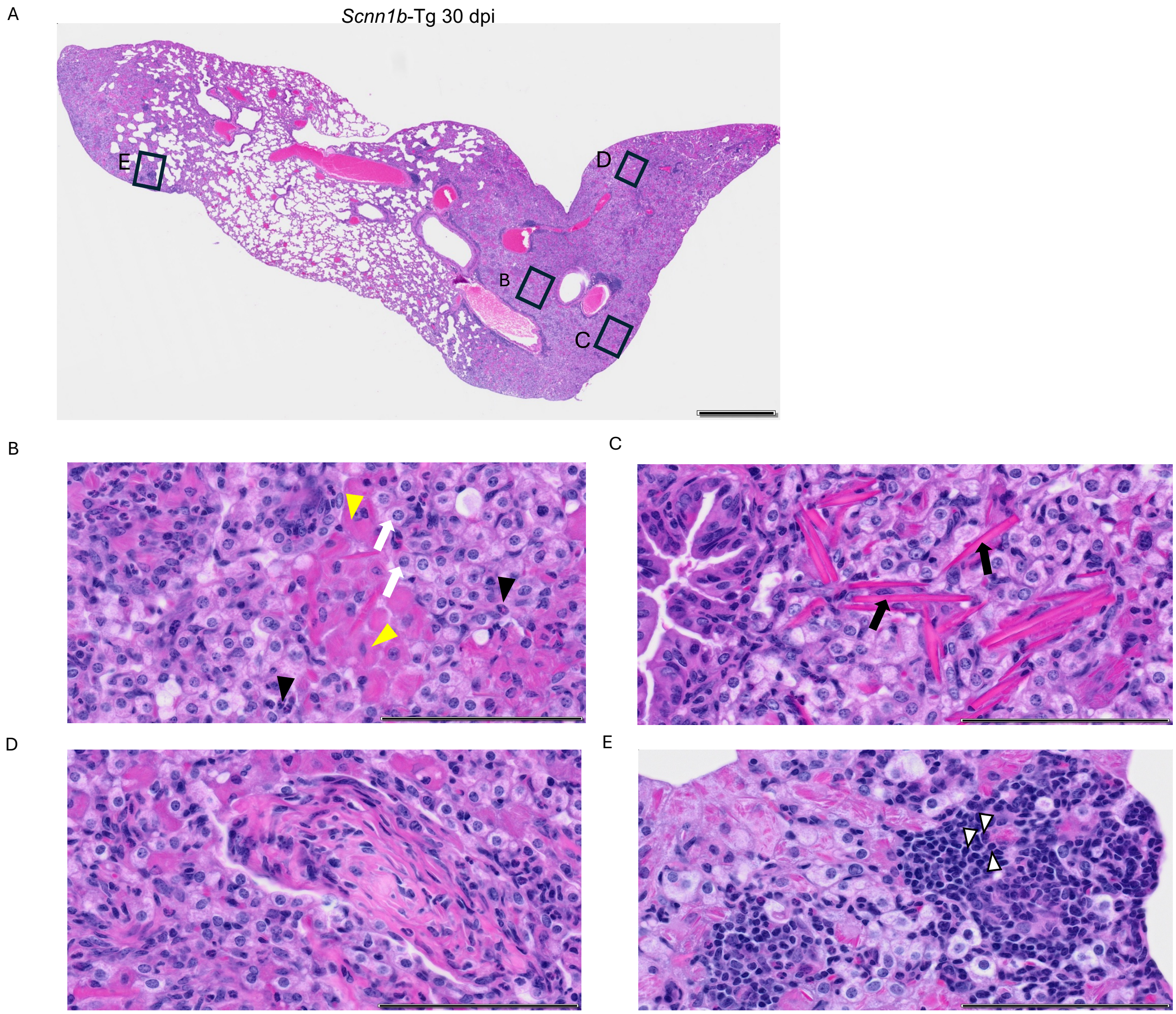

Figure S2: *Scnn1b*-Tg mice develop chronic lung disease following SARS-CoV-2 infection. (A) Low magnification H&E image of chronic lung lesion in *Scnn1b*-Tg mouse at 30 dpi. Scale bar 1 mm. (B-E) High magnification H&E images of chronic lung lesion in *Scnn1b*-Tg mouse at 30 dpi. Scale bar 50μm.(B) Alveolar epithelial cell hyperplasia, hyper-eosinophilic macrophages and eosinophils. (C) chitinase crystals. (D) Masson body. (E) Lymphoid aggregates. In B-E: white arrow: hyperplastic alveolar epithelial cell, black arrow-head: eosinophils, yellow arrow-head: hyper-eosinophilic macrophage, black arrow: chitinase crystals.

Figure S3

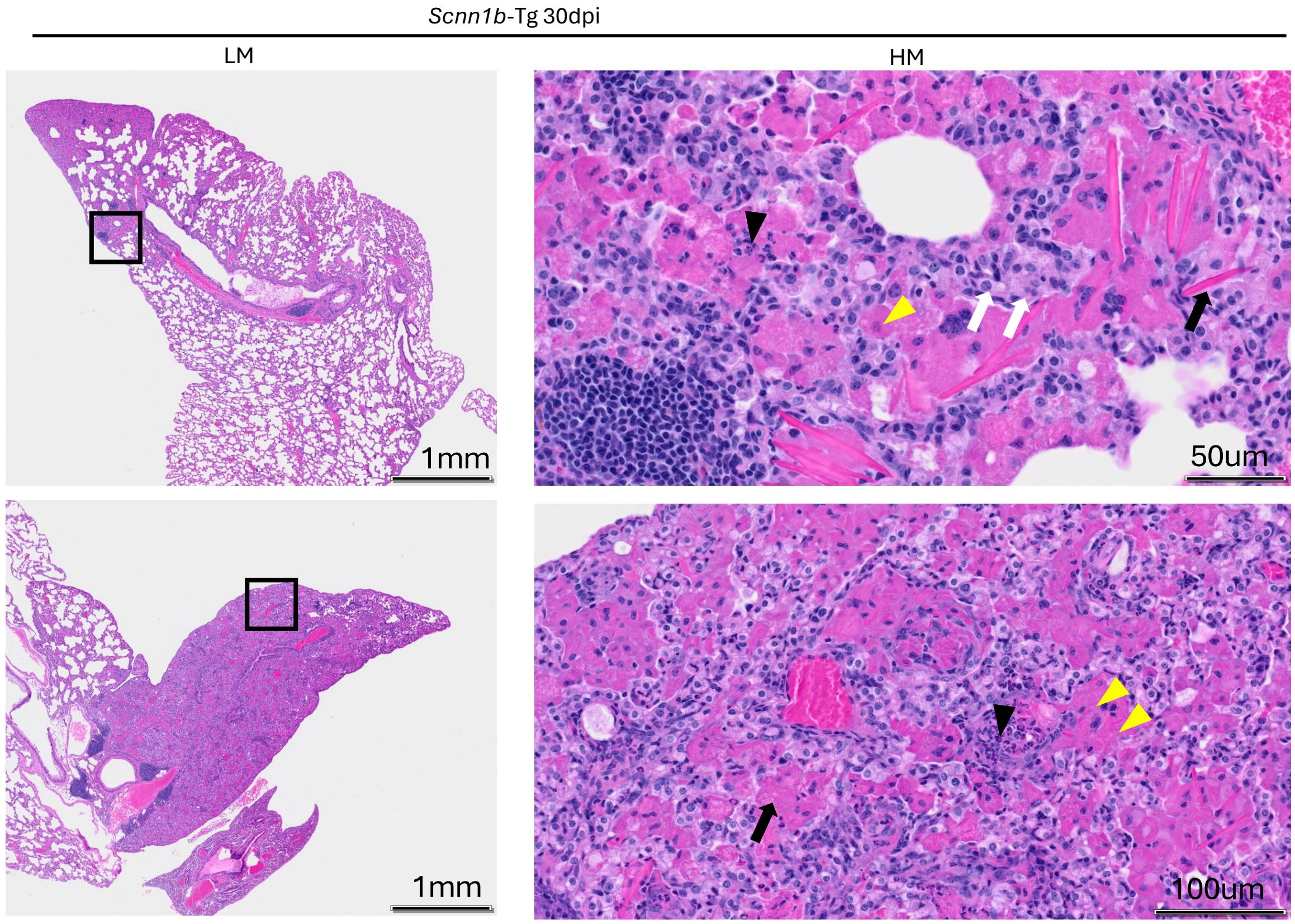

Figure S3: *Scnn1b*-Tg mice develop chronic lung disease following SARS-CoV-2 infection. H&E image of chronic lung lesion in *Scnn1b*-Tg mouse at 30 dpi. Demonstrated are: Alveolar epithelial cell hyperplasia, hyper-eosinophilic macrophages and eosinophils, chitinase crystals, Masson body and lymphoid aggregates. white arrow: hyperplastic alveolar epithelial cell, black arrow-head: eosinophils, yellow arrow-head: hyper-eosinophilic macrophage, black arrow: chitinase crystals.

Figure S4

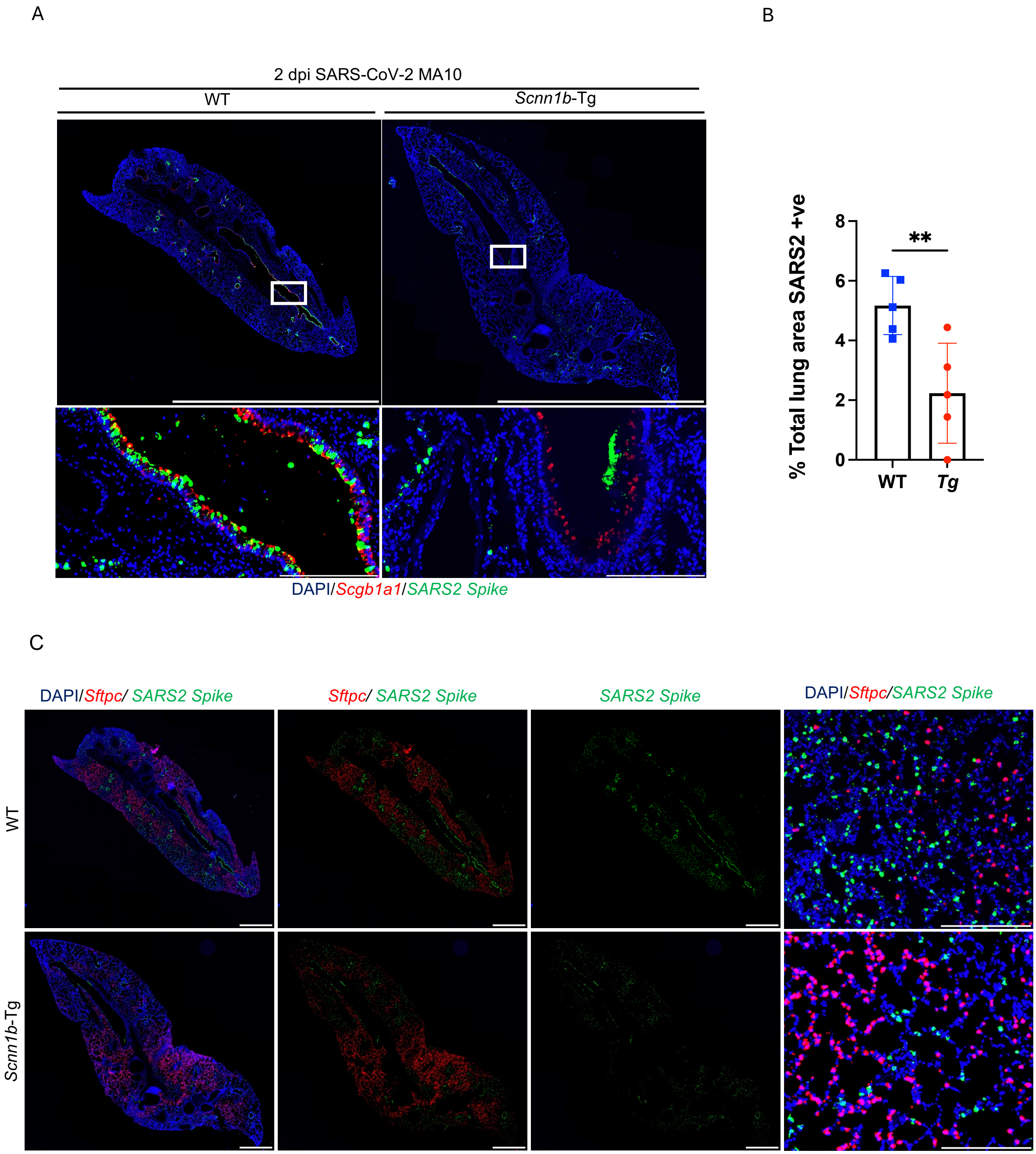

Figure S4: The airways of *Scnn1b*-Tg mice are protected from acute infection with SARS-CoV-2 MA10. (A) RNA-ISH of *Scgb1a1* (red) and SARS-CoV-2 spike (green) in WT and *Scnn1b*-Tg mice at 2 dpi. Scale bars represent 5 mm (low magnification) and 200 μm. (B) Quantitation of SARS-CoV-2 spike RNA-ISH signal, expressed as a percentage of total lung area. (C) RNA-ISH of *Sftpc* and SARS-CoV-2 spike in WT and *Scnn1b*-Tg mice at 2 dpi. Scale bars represent 1 mm (low magnification) and 200 μm.

Figure S5

A

		Baseline	2 dpi	30 dpi
Alveolar	Normal	WT=12, Tg=12		WT=12, Tg=12
	Infected		WT=12, Tg=12	
	Diseased			WT=12, Tg=12
Proximal Airway	Normal	WT=12, Tg=12		
	Infected		WT=12, Tg=12	
	Diseased			Wt=0, Tg=9
BADJ	Normal	WT=12, Tg=12		
	Infected		WT=12, Tg=12	
	Diseased			

B

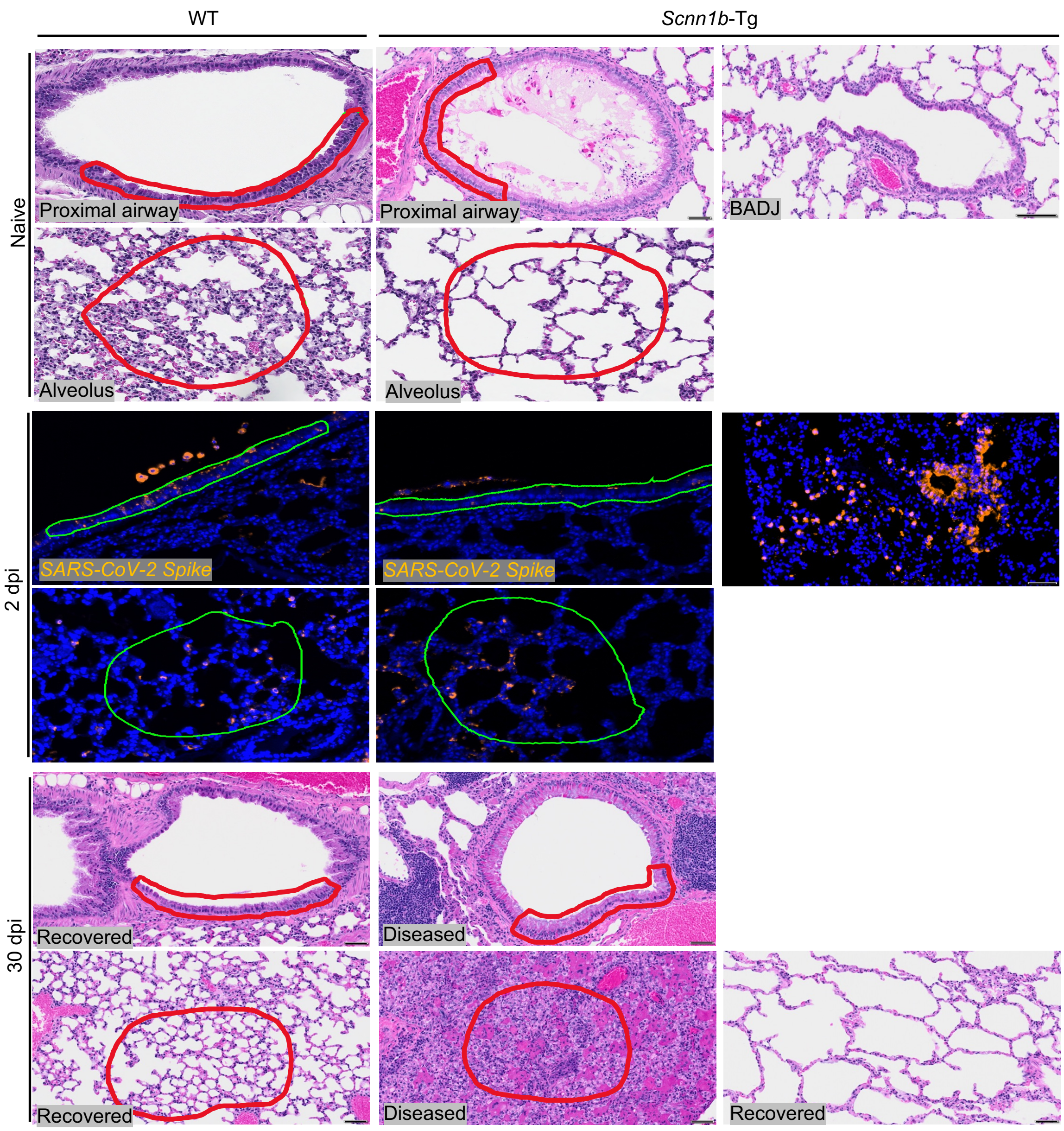

Figure S5: Transcriptional DSP experimental design and ROI selection. (A) Table outlining the ROI selection from anatomic site, disease state and time point. Number of ROIs. N=3 mice per group. (B) Example of ROI selection from naïve, 2 dpi and 30 dpi are shown. Top panels indicate selection of conducting airway (distal airway) epithelium; bottom panels show selection from alveolar regions. Selection for 2 dpi was based on RNA-ISH for SARS-CoV-2 Spike mRNA. For 30 dpi Scnn1b-Tg images, the ROIs selected from the chronic lesions (diseased) are shown, in addition to regions that appeared histologically normal 'recovered'.

Figure S6

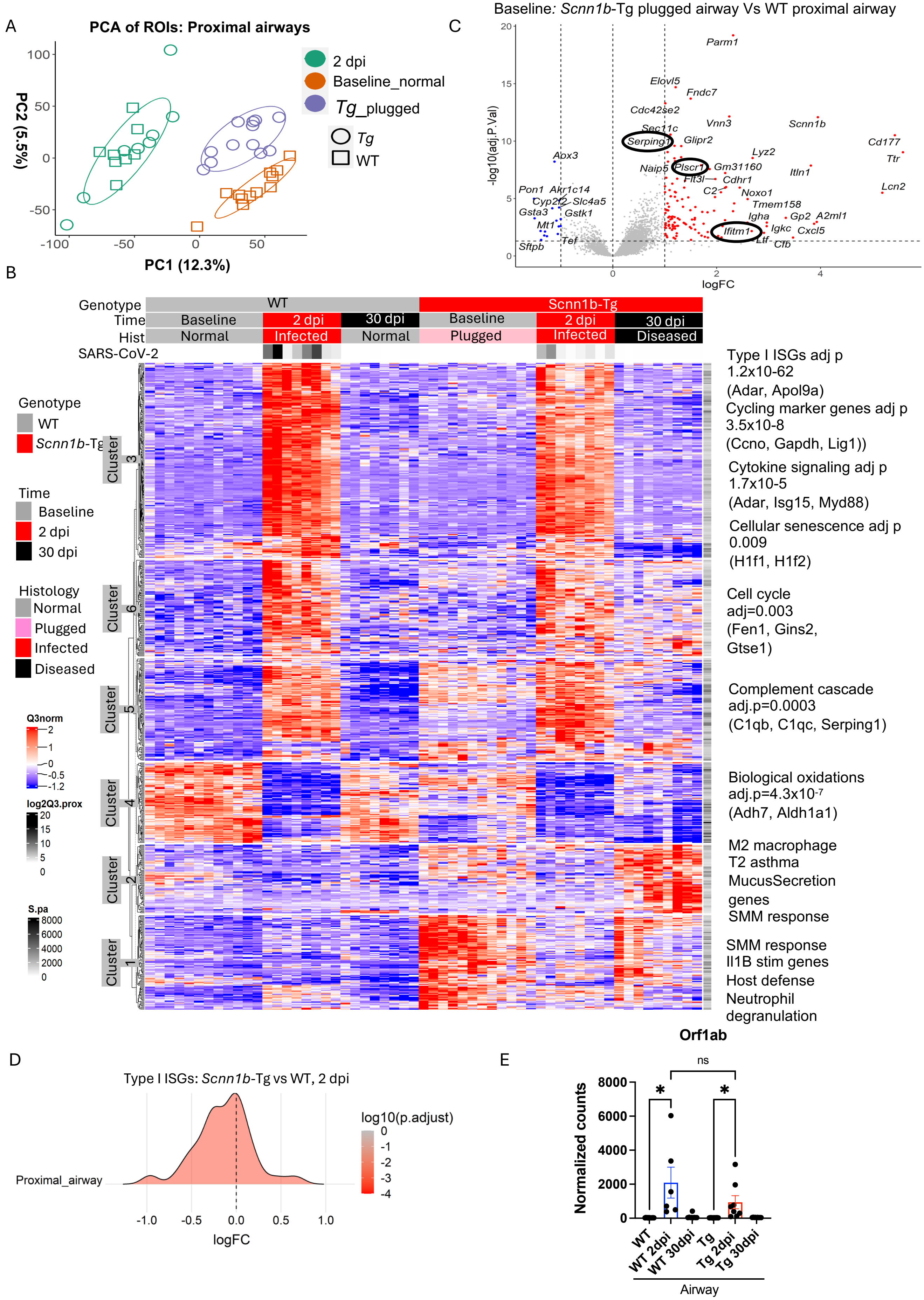

Figure S6: Transcriptional DSP analyses of proximal airways reveals primed airway epithelium in *Scnn1b*-Tg mice. (A) PCA of DSP analysis of proximal airways from WT and Scnn1b-Tg mice at baseline and 2 dpi. (B) DSP heatmap is shown for proximal airways across all timepoints and both genotypes. Selected pathways corresponding to the major clusters are shown. (C) Volcano plot of DEGs *Scnn1b*-Tg baseline proximal airways vs. WT baseline, proximal airways. Log fold change (logFC) on the x-axis and adjusted p-value on y-axis. Each dot represents a distinct gene. Red is upregulated in *Scnn1b*-Tg compared to WT, with logFC >1 and adj. p. val. <0.001. Blue; down-regulated in *Scnn1b*-Tg compared to WT. (D) A ridge plot of a Type I interferon gene set in *Scnn1b*-Tg proximal airways compared to WT proximal airways at 2 dpi indicating an overall downregulation of ISG responses in *Scnn1b*-Tg mice. (E) DSP Q3 normalized counts of virus gene *Orf1ab* in proximal airways over time course of infection. **** = p<0.00001.

Figure S7.

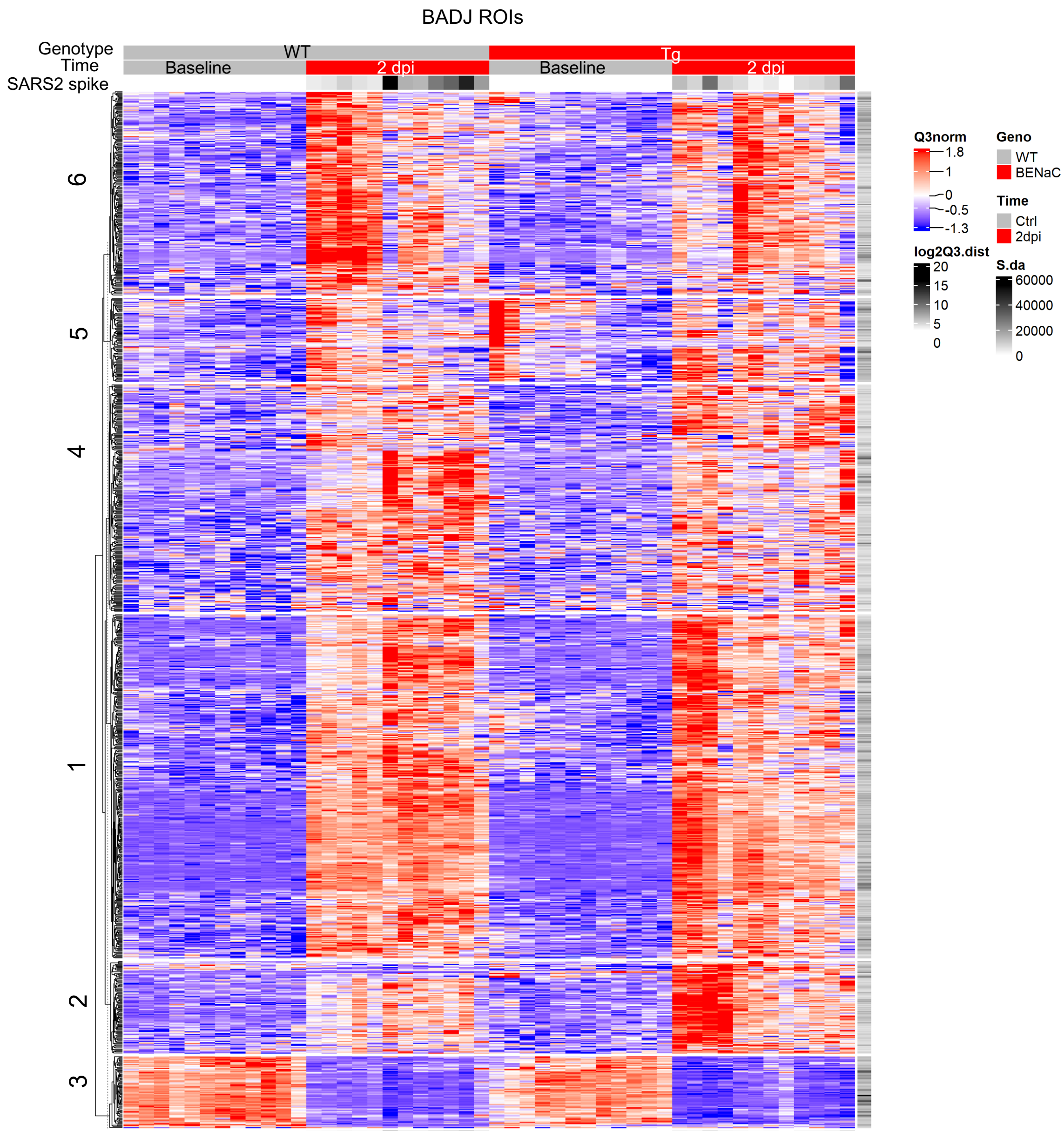

Figure S7: Transcriptional DSP analyses of BADJ reveals minimal between genotype differences.

Figure S8.

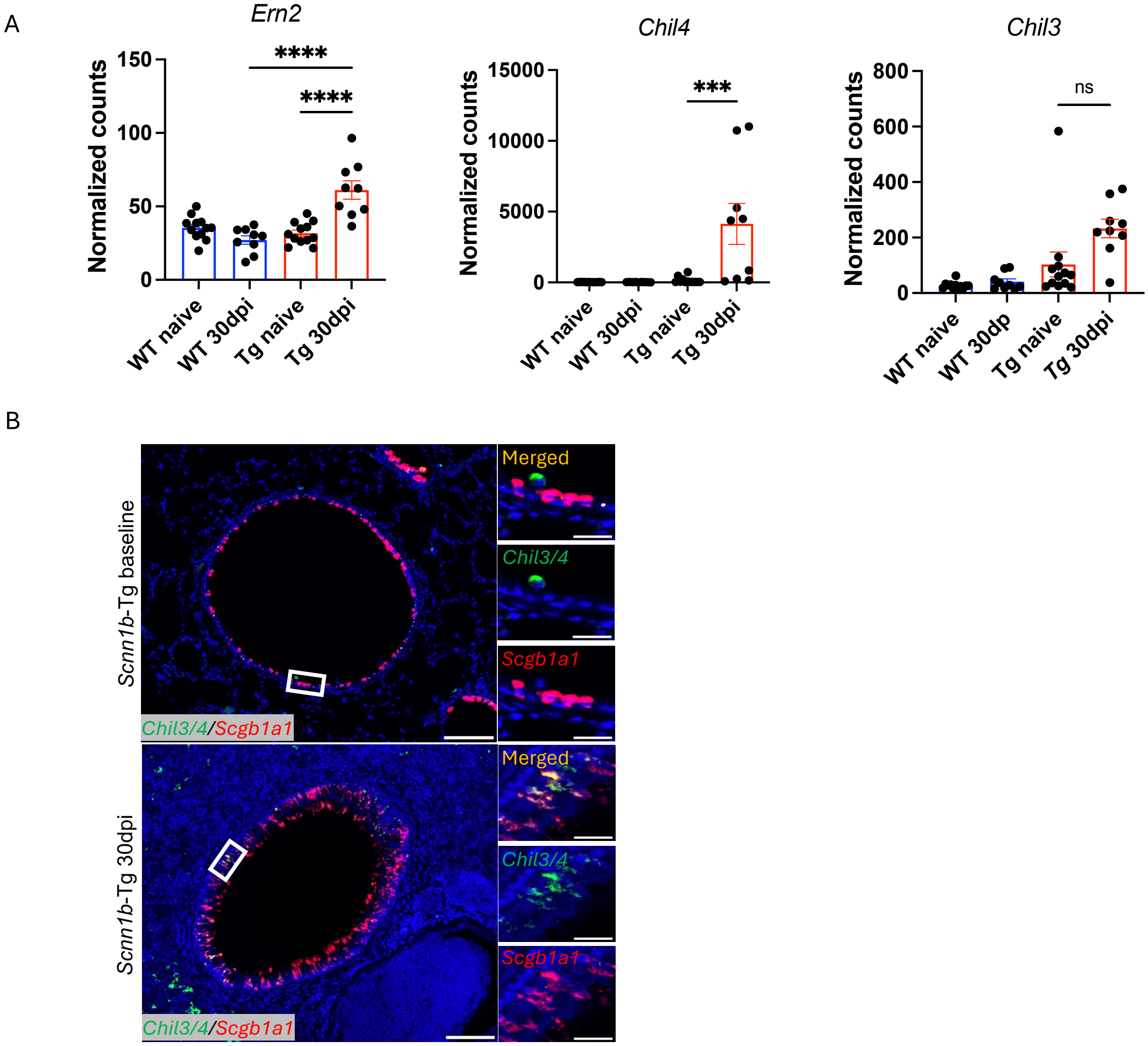

Figure S8: Markers of type 2 inflammation are present in the airways and lung parenchyma of *Scnn1b*-Tg mice 30 dpi with SARS-CoV-2 MA10. (A) DSP Q3 normalized counts of *Ern2* and *Retnla* from proximal airways of WT and *Scnn1b*-Tg mice in baseline conditions and after 30 dpi. (B)(i) RNA-ISH of *Chil3/4* and *Scgb1a1* in representative baseline and 30 dpi airway of Scnn1b-Tg mice. Inset, high power magnification with individual genes and merged. Scale bars represent 100μm (low magnification) and 20μm. (B) (ii) DSP Q3 normalized counts of *Chil4* and *Chil3* of proximal airways in WT and Scnn1b-Tg mice under naïve conditions and at 30 dpi. Paired t-test, if not shown was not tested.

Figure S9.

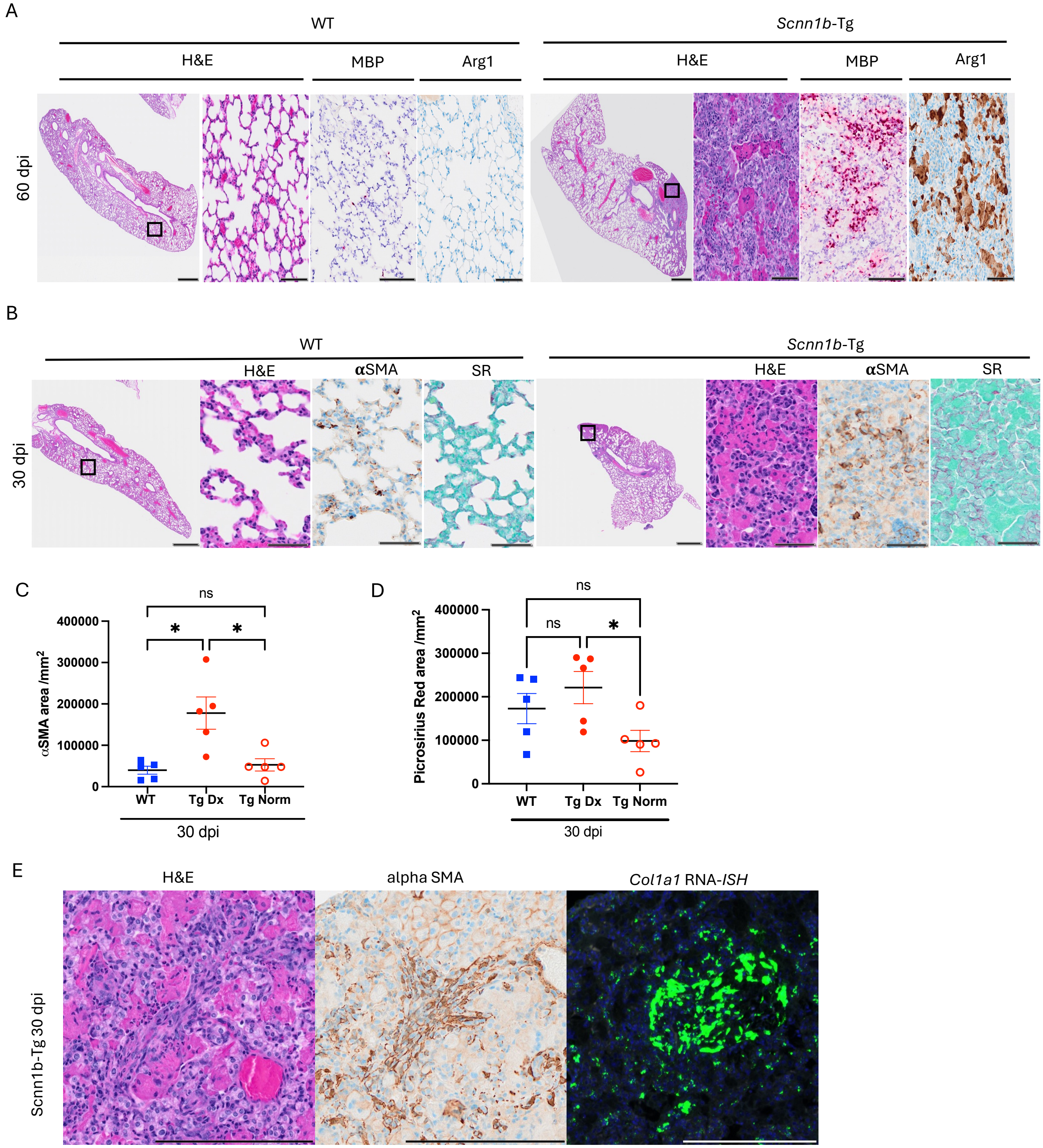

Figure S9: Myofibroblasts are increased in lungs of *Scnn1b*-Tg mice. (A) Histopathological analysis of lungs from WT and Scnn1b-Tg mouse at 60 dpi. H&E represents hematoxylin and eosin, MBP represents major basic protein, Arg1 represents arginase 1. Scale bars represent 1mm (low magnification) and 50μm. (B) Histopathological analysis of lungs from WT and *Scnn1b*-Tg mice. H&E represents hematoxylin and eosin, αSMA represent IHC for alpha-smooth muscle actin, SR represents picrosirius red staining. (C) Quantitation of αSMA IHC, and (D) quantitation of SR, normalized to total lung area. (E) Histopathological analysis of lungs from Scnn1b-Tg mouse at 30 dpi IHC for αSMA and RNA-ISH for Col1a1. One-way ANOVA, with Tukey's post test for multiple comparisons.

Figure S10.

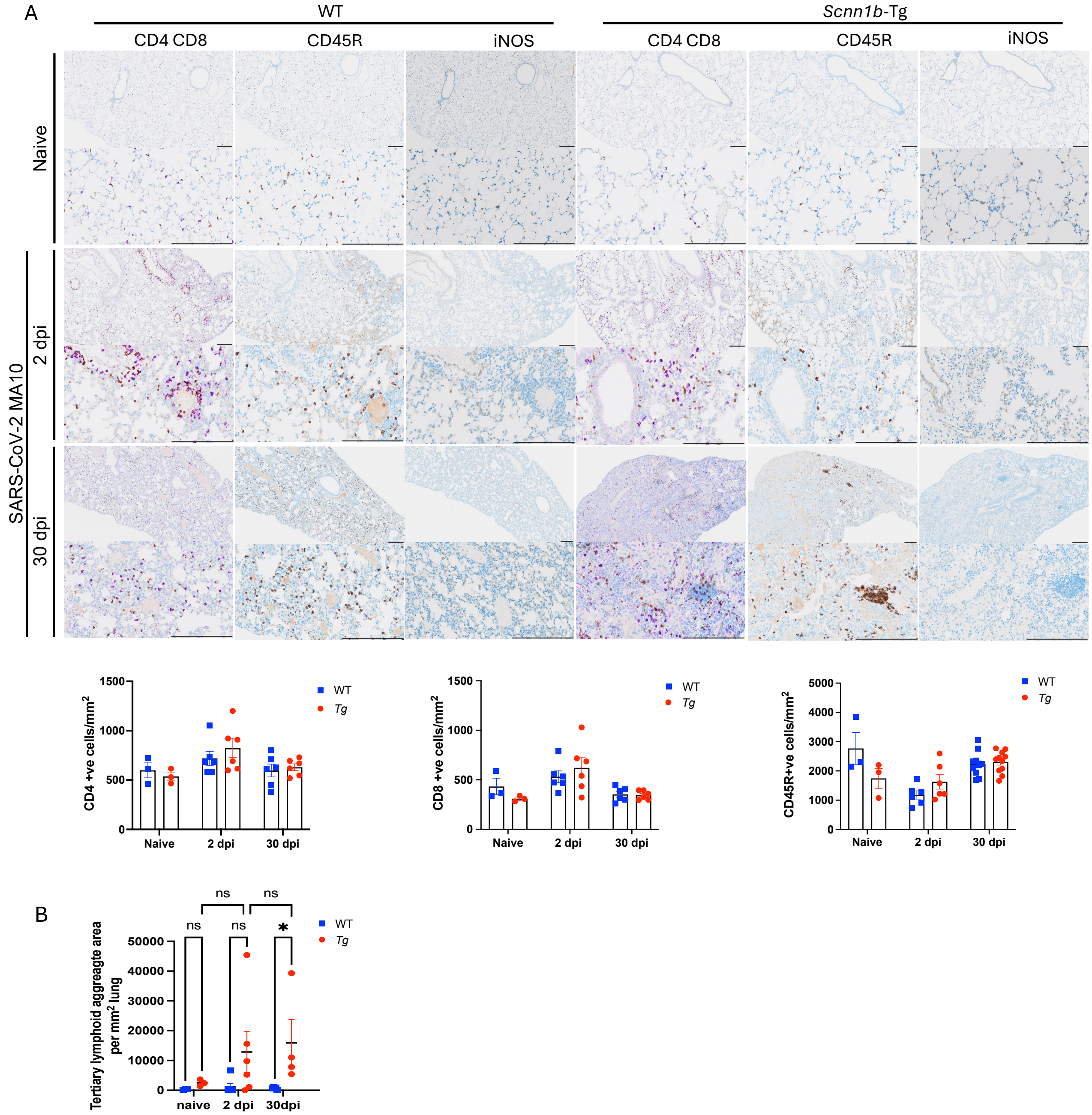

Figure S10: Lymphocytes are not increased in number in diseased lungs of Scnn1b-Tg mice at 30dpi but there is an increase in lymphoid aggregates. (A) Histopathological analyses of lungs from WT and Scnn1b-Tg mice over a time course following inoculation with SARS-CoV-2 MA10. IHC from CD4, CD8, CD45R, and iNOS. Scale bar represents 100μm (lower magnification) and 200μm. Quantitation of cell numbers for CD4, CD8, CD45R and lymphoid aggregates (B) are shown, normalized to total lung area.

Figure S11.

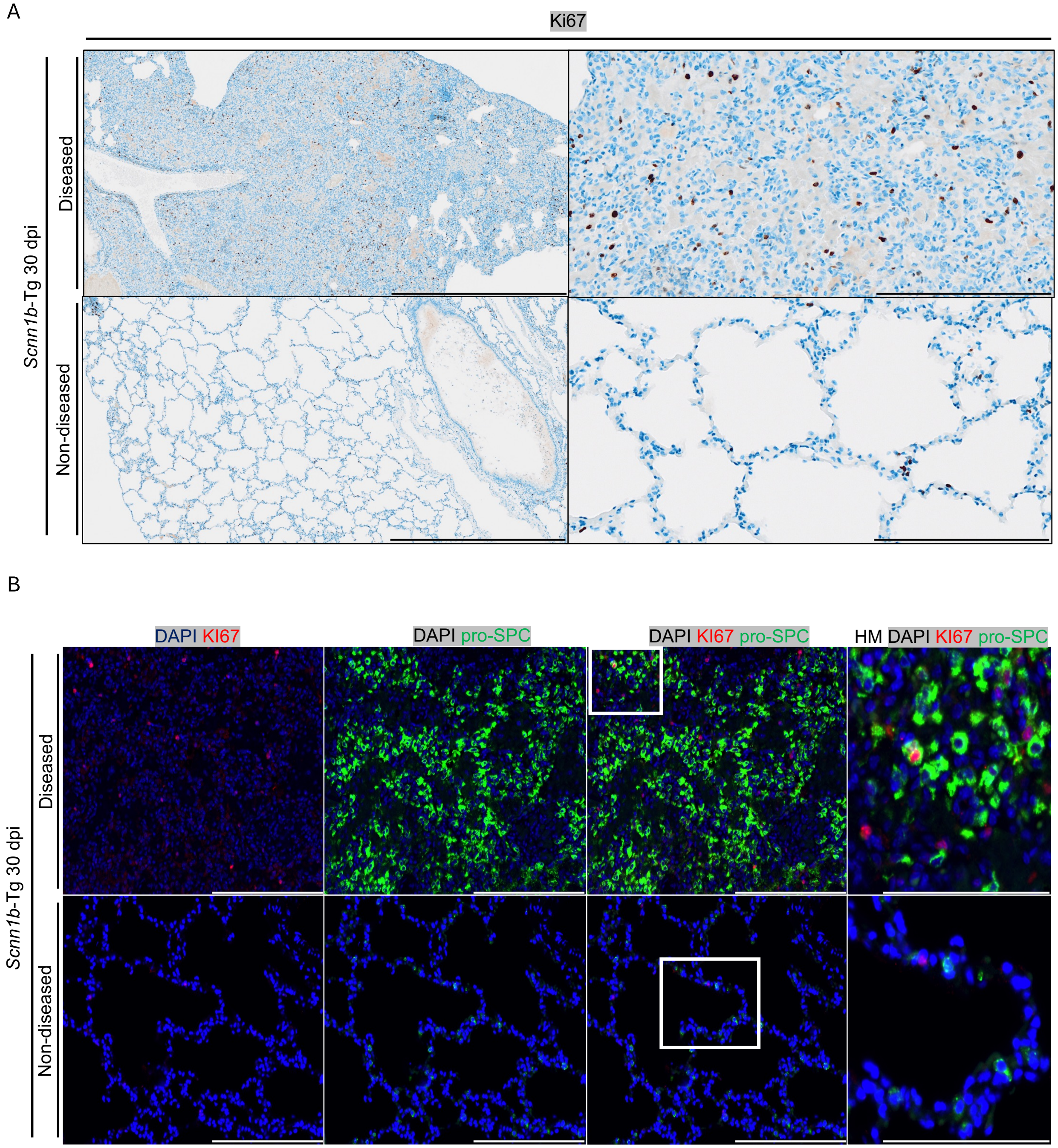

Figure S11: Proliferating cells are present in the diseased lungs of *Scnn1b*-Tg, including AT2 cells. (A) IHC-DAB (brown) of ki67. Scale bars represent 500μm (lower magnification) and 200μm. (B) IHC of ki67 and pro-surfactant protein C in *Scnn1b*-Tg mice at 30 dpi SARS-CoV-2 MA10 in diseased and non-diseased regions of the same lung. Scale bars represent 200μm and higher magnification (HM) 100μm.

Figure S12.

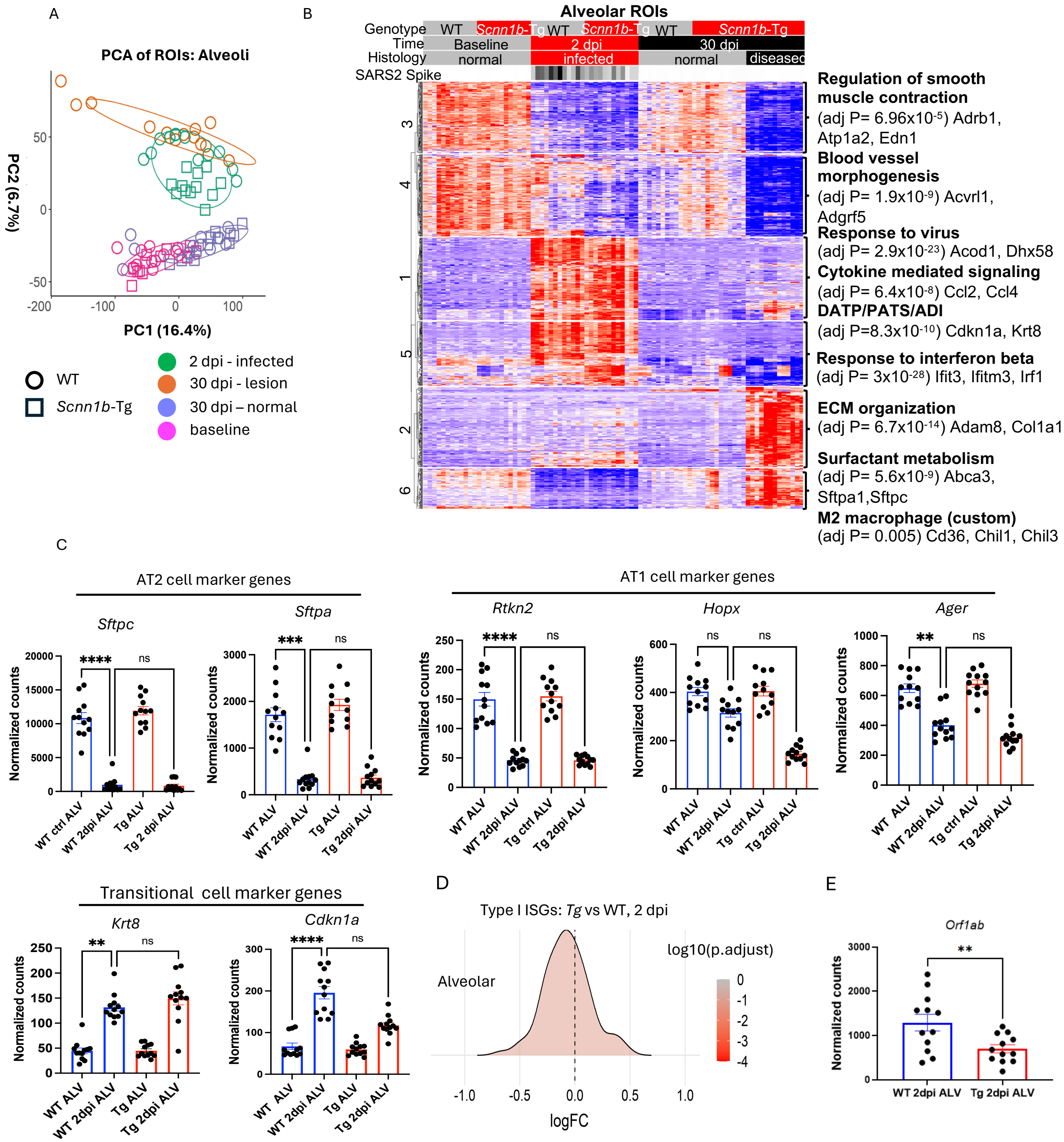

Figure S12: DSP reveals transcriptional changes in the alveolar compartment of diseased *Scnn1b*-Tg mice after SARS-CoV-2 MA10 infection. (A) PCA plot of alveolar regions are shown. All ROIs selected are displayed, with each color representing a disease state/time point and symbol representing genotype. (B) DSP heatmap is shown for alveolar ROIs across all timepoints and genotypes. Selected pathways corresponding to the major clusters are shown. (C) DSP Q3 normalized counts of AT2 cell marker genes, AT1 cell markers genes and transitional cell marker genes in alveolar regions over a time course post infection in WT and *Scnn1b*-Tg mice. (D) Ridge plot of Type I interferon gene set in *Scnn1b*-Tg alveolar regions compared to WT at 2 dpi (p=0.06). (E) DSP Q3 normalized counts of *Orf1ab* in alveolar regions at 2dpi in WT and *Scnn1b*-Tg mice.

Figure S13

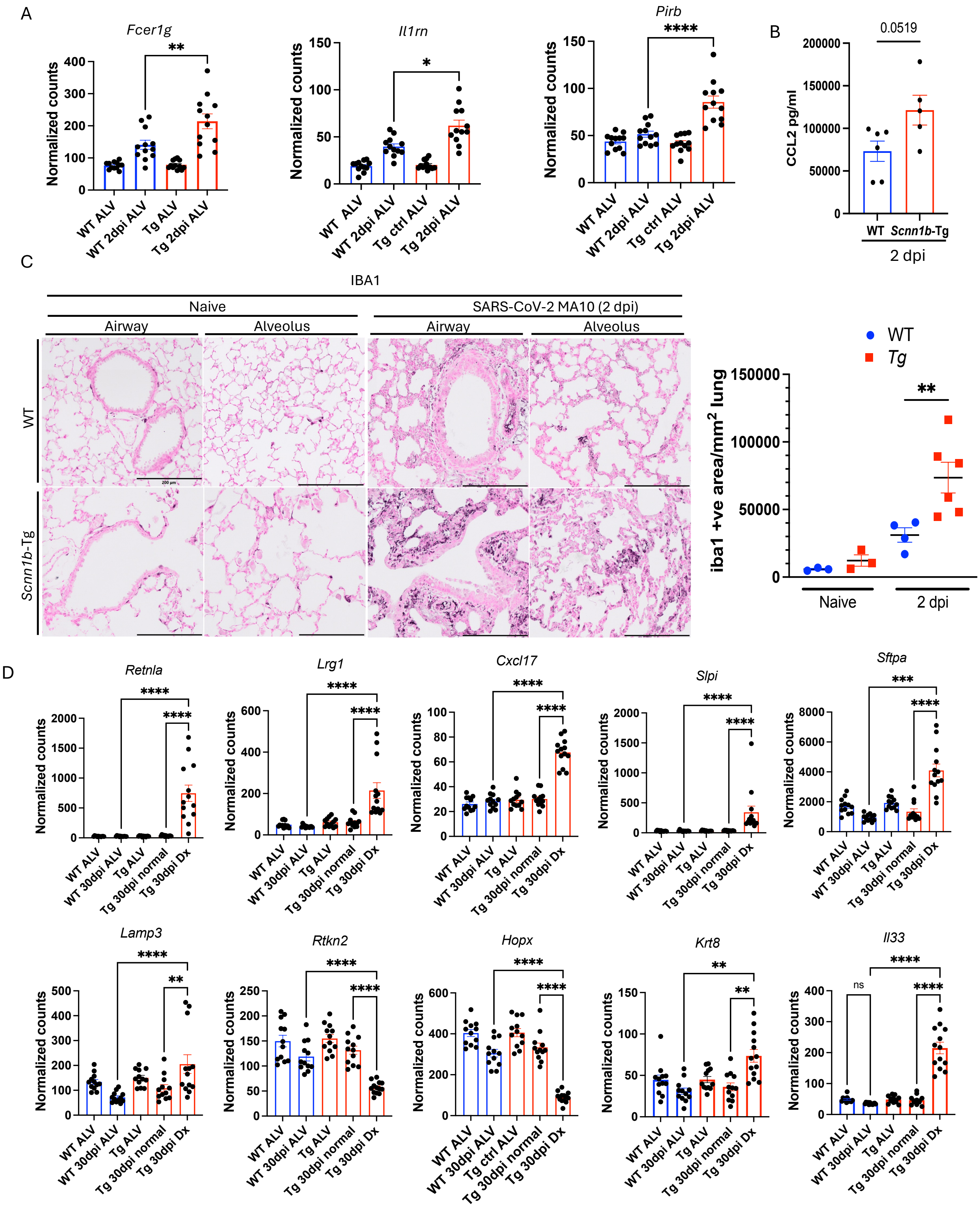

Figure S13: *Scnn1b*-Tg mice have an augmented inflammatory response in the alveolar compartment at 2dpi with SARS-CoV-2 MA10 and transcriptional evidence of activated AT2 cells at 30dpi. (A) DSP Q3 normalized counts of *Il1rn, Fcer1g, Tgfb1, Aif1* and *Pirb* over a time course post-infection in WT and Scnn1b-Tg mice. (B) CCL2 measured using a bioassay of homogenized lung tissue at 2 dpi for WT and Scnn1b-Tg mice. (C) IHC for IBA1 in the lungs of WT and Scnn1b-Tg mice, naïve and at 2dpi. Quantitation of IBA1 IHC, normalized to total lung area. (D) DSP Q3 normalized counts of *Retnla*, *Lrg1*, Cxcl17 and *Slpi* in alveolar region over a time course post infection in WT and Scnn1b-Tg mice. Dx=diseased lesions.

Figure S14

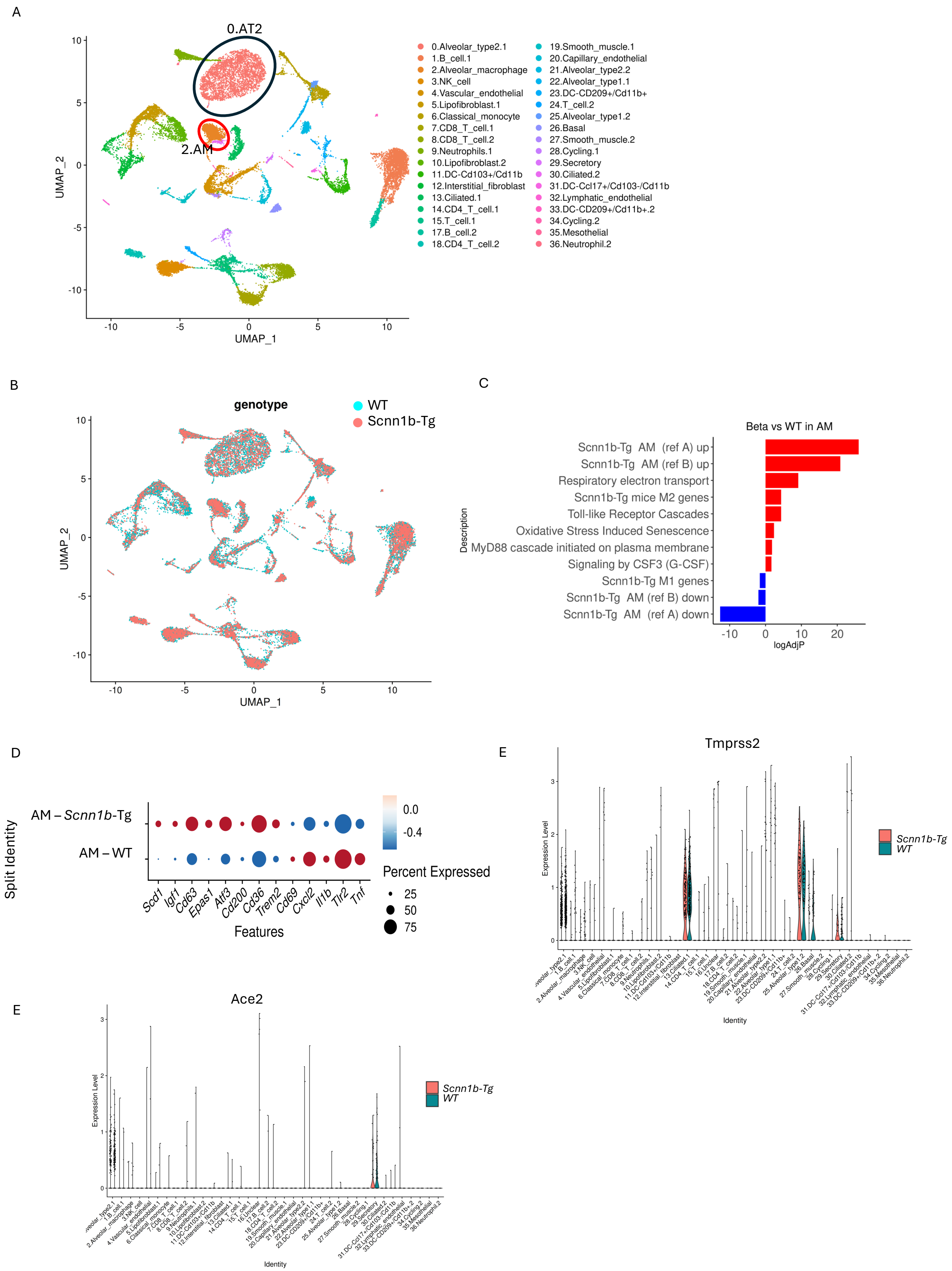

Figure S14: Single cell analyses reveals that AT2 cells and AM of baseline *Scnn1b*-Tg mice are transcriptionally distinct from WT. (A) Clustering of combined WT and *Scnn1b*-Tg into 37 distinct clusters by UMAP. (B) UMAP of all clusters with cells from WT highlighted in blue and Scnn1b-Tg in orange. (C) Custom pathways from single cell analysis of AM cluster comparing *Scnn1b*-Tg mice versus WT mice(Ref A = PMID:25204199, Ref B = PMID: 34764283). (D) Dot plot with markers of macrophage polarization. Dot size indicates percent expression and dot color average expression. (E) Violin plot of *Tmprss2* expression by cell type from scRNA-SEQ data. (F) Violin plot of *Ace2* expression by cell type from scRNA-SEQ data.

Figure S15.

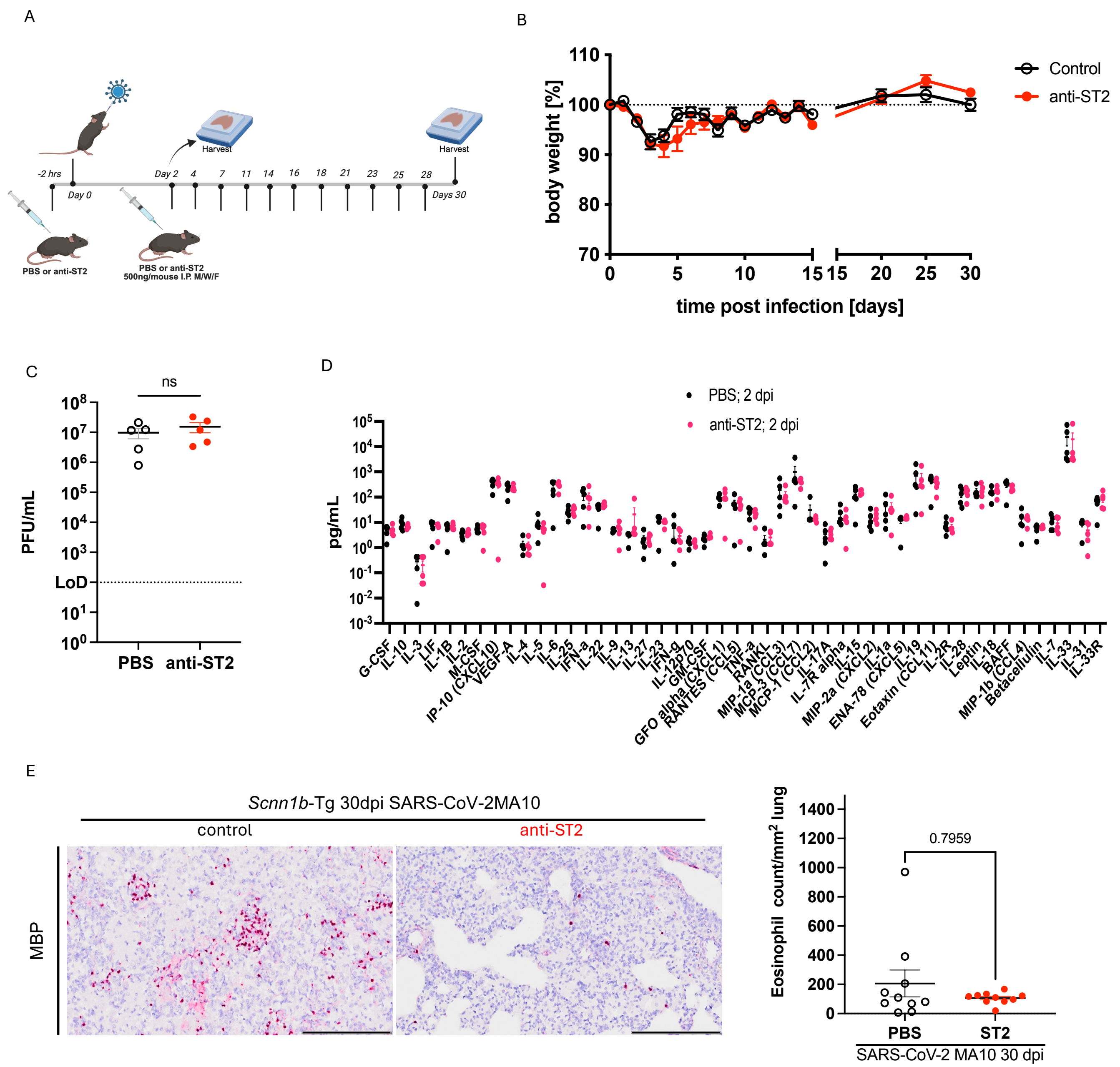

Figure S15: Treatment with an anti-ST2 monoclonal antibody abrogates the development of CEP in *Scnn1b*-Tg mice without impact on acute disease. (A) Experimental design. (B) Percent of starting weight over time course post inoculation with SARS-CoV-2 MA10. (C) Log-transformed infectious virus lung titers were assayed at 2 dpi with 104 PFU of SARS-CoV-2 MA10 virus. (D) Immunoassay of lung homogenates at 2 dpi from control mice and anti-ST2 treated mice. (E) IHC for major basic protein, MBP, in lung of mice treated with vehicle or anti-ST2 monoclonal at 30 dpi. Quantitation of eosinophil count per lung area based on MBP staining. Scale bars represent 100μm.

Figure S16.

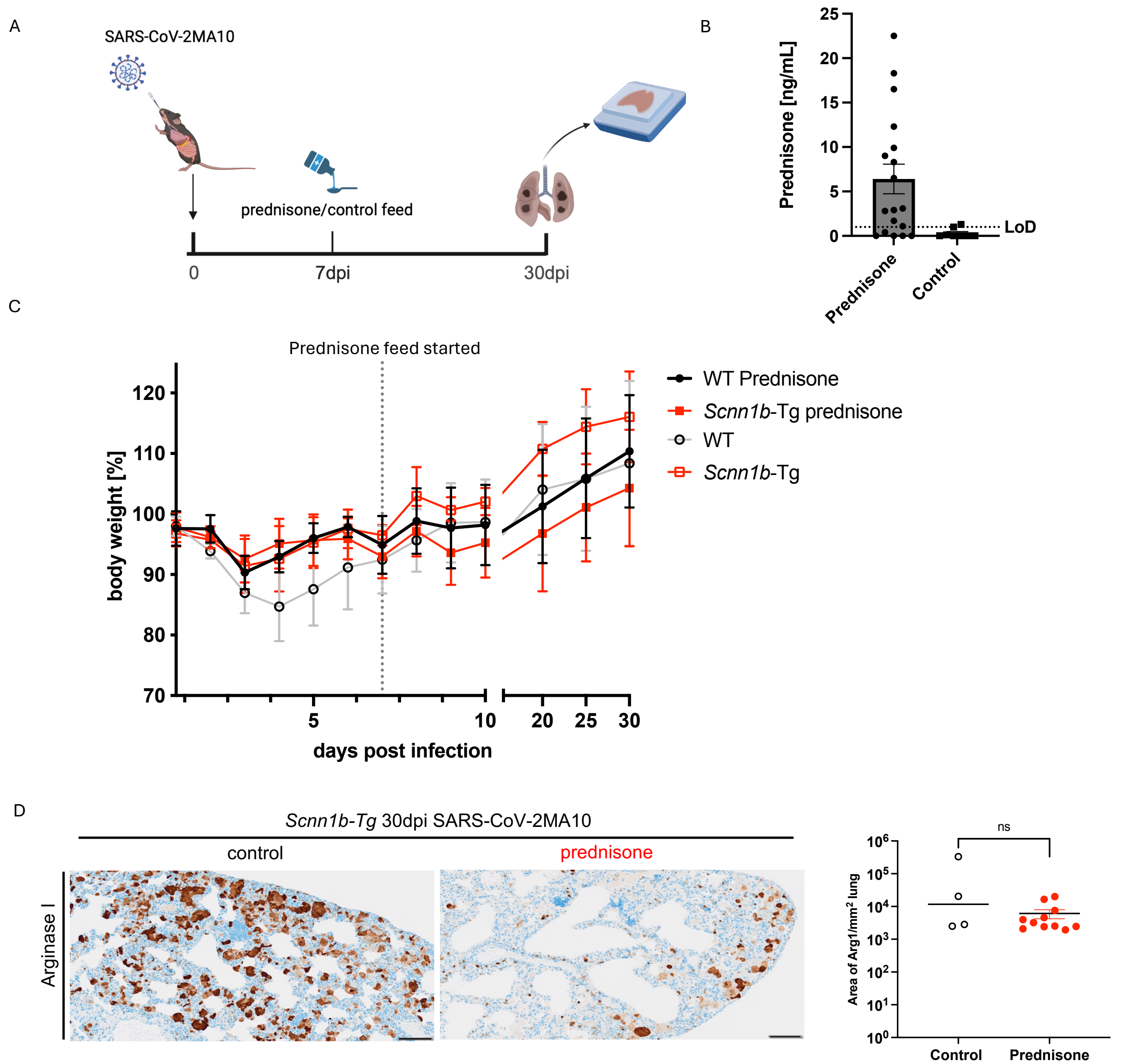

Figure S16. Treatment with prednisone abrogates the development of CEP in *Scnn1b*-Tg mice. (A) Experimental design. (B) Measurement of serum levels of prednisone using UHPLC-TOF mass spectrometry. (C) Percent of starting weigh over time course post inoculation with SARS-CoV-2 MA10. (D) IHC for Arginase 1 in lungs of *Scnn1b*-Tg mice receiving control diet or prednisone diet at 30 dpi. Quantitation of Arginase 1 area per lung area based on Arginase 1 IHC. Scale bars represent 100μm.
